# Integrated Ultrasound Characterization of the Diet-Induced Obesity (DIO) Model in Young Adult c57bl/6j Mice: Assessment of Cardiovascular, Renal and Hepatic Changes

**DOI:** 10.3390/jimaging10090217

**Published:** 2024-09-04

**Authors:** Sara Gargiulo, Virginia Barone, Denise Bonente, Tiziana Tamborrino, Giovanni Inzalaco, Lisa Gherardini, Eugenio Bertelli, Mario Chiariello

**Affiliations:** 1 Institute of Clinical Physiology, National Research Council, Via Fiorentina 1, 53100 Siena, Italy; giovanniinzalaco@cnr.it (G.I.); lisa.gherardini@cnr.it (L.G.); mario.chiariello@cnr.it (M.C.); 2 Core Research Laboratory (CRL), Istituto per lo Studio la Prevenzione e la Rete Oncologica (ISPRO), 53100 Siena, Italy; 3 Department of Molecular and Developmental Medicine, University of Siena, 53100 Siena, Italy; virginia.barone@unisi.it (V.B.); eugenio.bertelli@unisi.it (E.B.); 4 Department of Life Sciences, University of Siena, 53100 Siena, Italy; denise.bonente@student.unisi.it (D.B.); tiziana.tamborrino@student.unisi.it (T.T.)

**Keywords:** obesity, metabolic syndrome, animal model, C57Bl/6J mouse, high-frequency ultrasound imaging

## Abstract

Consuming an unbalanced diet and being overweight represent a global health problem in young people and adults of both sexes, and may lead to metabolic syndrome. The diet-induced obesity (DIO) model in the C57BL/6J mouse substrain that mimics the gradual weight gain in humans consuming a “Western-type” (WD) diet is of great interest. This study aims to characterize this animal model, using high-frequency ultrasound imaging (HFUS) as a complementary tool to longitudinally monitor changes in the liver, heart and kidney. Long-term WD feeding increased mice body weight (BW), liver/BW ratio and body condition score (BCS), transaminases, glucose and insulin, and caused dyslipidemia and insulin resistance. Echocardiography revealed subtle cardiac remodeling in WD-fed mice, highlighting a significant age–diet interaction for some left ventricular morphofunctional parameters. Qualitative and parametric HFUS analyses of the liver in WD-fed mice showed a progressive increase in echogenicity and echotexture heterogeneity, and equal or higher brightness of the renal cortex. Furthermore, renal circulation was impaired in WD-fed female mice. The ultrasound and histopathological findings were concordant. Overall, HFUS can improve the translational value of preclinical DIO models through an integrated approach with conventional methods, enabling a comprehensive identification of early stages of diseases in vivo and non-invasively, according to the 3Rs.

## 1. Introduction

Overweight and obesity, which can lead to age-associated disorders such as insulin resistance, dyslipidemia, non-alcoholic fatty liver disease (NAFLD), and cardiovascular diseases, have become a global health concern for the young and adult population, with gender-related differences. Consequently, preclinical models of obesity and metabolic syndrome are of great interest, particularly those that more closely resemble the gradual weight gain that occurs in Western humans due to excessive calorie consumption and unbalanced diets, leading to subtle obesity phenotypes. Although sexual dimorphism has been demonstrated in mouse models of obesity, little data are available on female mice [1].

Diet-induced obesity (DIO) in the C57BL/6J mouse substrain is widely used since it shows a high genetic predisposition to develop the metabolic disorders observed in obesity [1]. Furthermore, C57BL/6J mice are widely used to generate genetically modified animals, which could greatly help in understanding gene–disease interactions. In particular, the C57BL/6J strain from supplier Charles Rivers appears remarkably prone to obesity induced by both a high-fat diet (>60% energy from fat) and a “Western-type” diet (WD) (~45% energy from fat associated with higher levels of sucrose as an increased carbohydrate component) [2].

Some commercial rodent diets more closely resemble fat concentrations typically consumed by humans [3] and have been enriched with various levels of simple sugars (sucrose, fructose) and cholesterol to accelerate the progression of liver disease and induce atherosclerosis [4]. Overall, WD is able to induce obesity, hepatic steatosis and insulin resistance, fat deposition in peripheral tissues, and dysregulated lipid metabolism in rodent models [5], providing more relevant physiologic insight to study human metabolic syndrome [3,6].

Common study endpoints in DIO models include the following: (i) body weight and composition, (ii) food intake and feeding efficiency, (iii) blood chemistry parameters for the evaluation of liver and kidney function, (iv) lipid and glucose metabolism, (v) insulin resistance, (vi) hepatomegaly (measured as percentual liver to body weight ratio) and, (vii) histopathological alterations. In addition, high-frequency ultrasound imaging (HFUS) can be a useful complementary tool for studying the morphofunctional changes in the heart, liver and kidney in such animal models in vivo and non-invasively. Lessa and Resende et al. [7,8] first highlighted the feasibility of HFUS to study the progression of hepatic steatosis and/or fibrosis in rodent models of steatosis in female rats after 4, 8, and 15 weeks of liver injury induction and compared ultrasound findings to histological results [7]. In agreement with the literature, the authors found that an increased ratio of liver to right renal cortex echogenicity had a sensitivity of 90% and a specificity of 100% for the detection of fatty liver disease and was highly correlated with intermediate or severe histopathological scores. Furthermore, increased portal vein diameter was correlated with histological findings of hepatic steatosis and fibrosis and was indicative of portal hypertension [7]. Di Lascio and colleagues performed a detailed ultrasound evaluation of the cardiovascular system, liver, and kidneys in male ob/ob mice on SD and C57BL6 at 8 and 25 weeks of age, highlighting early cardiac dysfunction and vascular changes over time in ob/ob mice [9]. The same research group performed a wide phenotypic evaluation in male C57BL6 and db/db mice under SD at 14 weeks of age, including an extensive description of cardiovascular, renal, and hepatic ultrasound parameters [10]. They highlighted not only higher body weight (BW), blood triglycerides, and aspartate aminotransferase (AST) values in db/db mice, but also differences in structural and functional features of the left ventricle, as well as of the abdominal aorta, carotid artery, and renal vessels [10]. The ratio between liver and renal echogenicity showed a higher trend in db/db mice without reaching statistical significance. The authors emphasized that the overall results of their study could be a useful reference for future intervention experiments using these specific mouse strains and that their imaging approach could be useful to longitudinally characterize different mouse models in the field of obesity research [9,10]. Similarly, Cui and colleagues explored the utility of combining ultrasound imaging and serum biomarkers to detect steatohepatitis and fibrosis in male C57L/J mice after treatment with diethylnitrosamine and a high-fat diet. Animals were serially monitored by ultrasound from 1 to 10 months of age, and imaging results were related to anatomical and histopathological findings. Overall, the authors highlighted the translational utility of a complementary approach that combines the conventional assessment of serum parameters and liver echostructure to improve the accuracy of liver pathology information [11]. More recently, Pantaleão and coworkers focused on the ability of ultrasound to detect early alterations of NAFLD in WD-fed male rats [12]. They analyzed the relationship between liver and renal cortex echogenicity at different time points over 16 weeks, integrating body and liver weight measurements and anatomo-histological findings. WD-fed rats had a significantly higher liver weight and liver-to-renal brightness ratio than controls, with histology supporting the ultrasound findings. Overall, they highlighted the translational value of the WD-induced NAFLD and the proposed HFUS approach in assessing the early stages of liver disease in rats, providing baseline knowledge for further investigations in similar animal models. The present study had two purposes. One was to extensively phenotype the C57BL/6J mouse strain as a DIO model paradigm in order to collect baseline data for further comparison with mice with similar genetic backgrounds and engineered for specific genes of interest. The other was to evaluate the utility of HFUS as a complementary tool to detect early and monitor over time subtle morphofunctional changes in the liver, heart, and kidney. The novelty of the present work, compared to previous studies using HFUS in similar contexts, concerns the following: the DIO animal model (C57BL/6J strain from Charles Rivers, WD); the investigation of both genders; the timeline of metabolic syndrome monitoring (early stages of the disease in young and adult mice); the state-of-the-art ultrasound methods used to analyze liver changes; broad-spectrum parameters monitored over time in the same subject, useful to better clarify the complex physiological effects of dietary interventions according to the 3Rs.

## 2. Materials and Methods

### 2.1. Compliance with Ethical Standards

The experimental procedures complied with the European Communities Council directive (2010/63/EU) national regulations (D.L. 26/2014). The present study was approved by the Animal Welfare Board of Fondazione Toscana Life Sciences and by the Italian Ministry of Health (authorization number 175/2021-03-03 and 175/2023-02-17). The Animal Research: Reporting of In Vivo Experiments (ARRIVE) guidelines and National Institutes of Health (NIH) recommendations were followed. All efforts were made to minimize the number of animals used and their suffering. All the study data are maintained and available upon request from the corresponding author.

### 2.2. Study Design and Animals

C57BL/6J mice were purchased from Jackson Laboratories (Bar Harbor, ME) via Charles River (Calco, LC, Italy) at 7 weeks of age. Mice were housed in groups of up to 4 mice per cage (individual housing could produce bias in metabolic murine models) under standard conditions (12 h light cycle, room temperature of 20–23 °C), and free access to food and water [13]. After 7 days of acclimation, the animals were marked for identification. For practical reasons, we divided the 32 experimental animals into two cohorts subsequently tested as diet groups (WD; SD), each containing 16 mice and divided into two sets based on sex (eight males and eight females), examined simultaneously. Experimental sets of mixed sexes are not recommended for studies of energy balance due to sex differences in adipose tissue depots, inflammatory cytokines, blood lipid profile, and insulin activity [14,15].

Mice in the DIO group were switched from the standard diet (SD: 3% fat, 4RF21, Mucedola^®^, Milan, Italy; Kcal 18.5% from protein; 3% from fat; 53.5% carbohydrates, 3% sucrose; kcal/g 3.150) to a lipid-rich diet (WD: 0.2% cholesterol and 21% butter, Western U8958 version 35, SAFE^®^, Paris, France; Kcal 14.4% from protein; 38.1% from fat; 47% carbohydrates, 33% sucrose; kcal/g 4.2594) starting from the beginning of 8 weeks of age until the end of 24 weeks of age. The WD was stored at 4 °C and replaced once per week to avoid lipid peroxidation. The global feeding period was selected based on the literature considering findings in mice fed a high-fat diet [16,17]. All WD-fed mice appeared healthy and active throughout the diet intervention period, and no mouse had to be euthanized before 24 weeks of age.

Mice in the control group were fed SD from weaning until 24 weeks of age. One male mouse was excluded from the study for dental malocclusion. Body weight and food consumption were monitored twice per week using a precision balance reading to the nearest 0.1 g. Mice were weighted immediately after food replenishment [18]. The amount of food remaining in the cages was subtracted from the amount initially recorded, including any large (>5 mm) pieces of uneaten food found on the cage floor [18]. For each mouse, average daily food intake (g) per week was calculated. According to NIH-MMPCs guidelines, we calculated the body weight (BW) change (g) from the initial body weight measurements to analyze the overall effect of diet on BW [15]. Furthermore, the body condition score (BCS), a non-invasive method for assessing health and nutritional status in laboratory rodents, was monitored at relevant time points [19,20]. Food intake of mice was analyzed as the daily amount of food (g) consumed by a single mouse for each experimental week. To evaluate changes in metabolism according to food intake, the food efficiency ratio (FER) was monitored weekly as the (total weight gain/total food intake) × 100. As recommended, data were analyzed separately by sex, due to metabolic differences related to hormonal influence, and stress was minimized by assigning responsibility for animal handling to a single, experienced Doctor of Veterinary Medicine (DVM) throughout the study [15]. Experimental blinding was not achieved because the sole experimenter and sonographer inevitably knew the housing condition and the dietary regimen of each mouse.

### 2.3. Biochemical Analysis

Sample collection time of day, feeding state, route, and vessels for blood collection were standardized throughout the study. At 8 and 16 weeks of age, whole blood was collected in K3-EDTA vials (100 µL/mouse, at 8:00–9:00 a.m.) by facial vein puncture (40–60 s), under 3% isoflurane + 2Lt/min oxygen anesthesia consistent with current veterinary recommendations [21,22]. Non-fasting values of glucose, cholesterol, and triglycerides were measured (Multicare IN, Biochemical System International), and hyperglycemia was monitored as non-fasting blood glucose ≥ 250 mg/dL [23,24].

At 24 weeks of age, mice were fasted for 3 h at 8:00–11:00 a.m. and blood was collected prior to euthanasia under 3% isoflurane + 2Lt/min oxygen anesthesia by cardiac puncture (40–60 s) [25]. Blood samples were centrifuged (3500 rpm, 15 min) and sera were immediately frozen (−20 °C). Serum alanine aminotransferase (ALT), aspartate transaminase (AST), cholesterol, triglycerides, glucose, and blood urea nitrogen (BUN) results were provided by the laboratories of a research organization (IZSLT, Rome, Italy; Galileo Research, Pisa, Italy) accredited by the Italian Ministry of Health to carry out studies on rodents for research purposes. In addition, fasted serum insulin was determined using a Mercodia ultrasensitive mouse insulin ELISA kit. The insulin ELISA protocol is adopted from the manufacturer’s instructions. Insulin units were converted from ng/mL to pmol/L by multiplying by 172.18, and subsequently to µIU/mL by dividing by a conversion factor of 6. Measured blood glucose was converted from mg/dL to mmol/L by multiplying by 0.0555. HOMA-IR was calculated by multiplying insulin (µIU/mL) by glucose (mmol/L) and dividing by 22.5. The translational calculation of HOMA-IR adjusted to murine species was adopted from Fraulob et al. (2010) [26], and the conversion factor of insulin was derived from Knopp et al. (2019) [27]. Alternatively, insulin sensitivity was assessed by using the homeostasis model assessment-2 (HOMA2) index using an online-based calculator version 2.2.3 on the Diabetes Trials Unit of the University of Oxford website [28], providing values for insulin resistance (HOMA-IR), steady-state β-cell function (HOMA-%B), and insulin sensitivity (HOMA-%S) [29].

The metabolic changes in the blood chemistry parameters in C57BL/6J have been evaluated against gender- and age-dependent reference intervals [30,31,32].

### 2.4. Ultrasound Imaging

Ultrasound images at 8, 16 and 24 weeks of age were acquired and analyzed by an experienced veterinary operator, using a high-frequency ultrasound unit (VisualSonics 2100, Toronto, ON, Canada) equipped with an MS550 Blue Transducer probe (central frequency 40 MHz; focal length 6 mm; depth of penetration 5–15 mm; 30–40 µm axial and 70–90 µm lateral resolution), and the Vevo Lab software (version 3.0.0). Mice were not fasted before imaging to minimize discomfort and avoid fasting-induced metabolic changes [33]. Body temperature was monitored using a rectal probe and kept in a physiological interval by an infrared lamp. During imaging sessions, mice were kept under inhalant anesthesia (induction chamber: 4% isoflurane plus 2 Lt/min oxygen; maintenance with nose cone: 1.5–1.8%) on a heated platform. These concentrations of isoflurane are recommended for cardiac function studies because they produce stable body temperature, mean arterial pressure (MAP), and heart rate (*hr*) values in C57BL/6 mice comparable to those observed in awake animals [34]. The induction of anesthesia and stabilization of heart rate lasted approximately 10 min before echocardiography [35]. Approximately 5 min of this time interval was useful for performing trichotomy of the thorax and abdomen of mice by depilatory cream. In that way, the experimental procedure was refined to prevent handling stress and health risks resulting from the ingestion of depilatory creams during normal grooming behavior [36]. Next, mice were positioned in dorsal recumbency, a coupling gel was applied to the depilated skin, and the animal’s limbs were coated with conductive paste and taped to four electrocardiogram (ECG) electrodes incorporated in the platform for the measurement of heart rate, ECG and respiratory rate. Echocardiography took no more than 10 min per mouse. To be consistent between animals and images, we stored optimized acquisition settings on the instrument. All B-mode ultrasound images were acquired under the same data capture settings (frequency = 40 MHz, frame rate = 20 images/s, gain = 30 dB, depth = 11 mm, width = 13.00 mm, dynamic range = 60 dB, line density = high, sensitivity = high) for each mouse. The time gain compensation was set to adjust the tissue echogenicity to be as constant as possible regardless of the depth, and the transmit power was set at 100%. Two-dimensional echocardiography B-mode loops were acquired through the chest wall in single-plane parasternal long-axis (LAX) and short axis (SAX), and subsequently analyzed offline. The following measurements of the left ventricle (LV) were performed in LAX: the thickness of the interventricular septum (IVS) or the LV anterior wall (LVAW); the LV interior diameter (LVID); and the LV posterior wall (LVPW). End-diastolic (d) and end-systolic (s) measurements were obtained at the time of maximal and minimal internal chamber dimensions, respectively, and were measured for at least three heartbeats to calculate an average value [37,38,39].

These measurements were used to calculate:

- Ejection fraction (EF, %): 100 ∗ ((LV Vol;d − LV Vol;s)/LV Vol;d) as a measurement of how much blood the left ventricle pumps out with each contraction;
- Fractional shortening (FS, %): 100 ∗ ((LVID;d − LVID;s)/LVID;d) as a measurement of the reduction in the length of the end-diastolic diameter that occurs by the end of systole;
- LV mass (g) corrected (corr): 1.053 ∗ ((LVID;d + LVPW;d + IVS;d)^3^ − LVID;d^3^) ∗ 0.8 considering myocardial density approximately 1.053 g/mL and multiplying the LV mass value by 0.8 to “correct” for an overestimation of LV mass, according to the manufacturer’s instructions [40].
- LV volume (vol); d (µL): ((7.0/(2.4 + LVID;d)) ∗ LVID;d^3^
- LV vol; s (µL) ((7.0/(2.4 + LVID;s)) ∗ LVID;s^3^
- Stroke volume (SV) (µL): (LV Vol, d − LV Vol, s) as the volume of blood pumped out of the left ventricle of the heart during each systolic cardiac contraction;
- Cardiac output (CO) (mL/minute): (SV × *hr*)/1000 as the amount of blood the heart pumps through the circulatory system in a minute.
- Relative wall thickness (RWT): as the index of LV geometric remodeling less vulnerable to variability related to body morphometry, calculated as septal wall thickness + posterior wall thickness divided by LV diastolic diameter ((SWTd + PWTd)/LVEDD)) [39,40,41,42,43]

Based on the recommendation about cardiac measures indexing [39,40,41,42], 1D measures (diameter) were normalized by BW^1/3^, and 3D measures (volume/mass) were normalized by BW to avoid biases related to BW changes [43].

Next, liver images were acquired by two-dimensional B-mode imaging through the ventral body wall and subsequently analyzed offline. Four to six B-mode cine-loops imaging the left and right lobes of the liver and at the caudate lobe–right kidney interface were acquired in sagittal and axial imaging planes. To be consistent between animals and images, all B-mode ultrasound images were acquired under the same data capture settings (frequency = 40 MHz, frame rate = 16 images/s, gain = 30 dB, depth = 11 mm, width = 13.00 mm, dynamic range = 60 dB, sensitivity = high) for each mouse. The time gain compensation was set to adjust the tissue echogenicity as constant as possible regardless of the depth, and the transmit power was set at 100%. The liver parenchyma was examined for echogenicity, echostructure, presence or absence of nodules, and border definition. B-mode images (256 grayscale values) were selected at the tele-expiratory phase to reduce artifacts related to respiratory motion to a minimum, as well as shadowing artifacts related to bone or intestinal gas.

The detection and grading of hepatic steatosis by visual inspection and parametric evaluation of the liver were performed according to previously validated protocols in humans, already applied to mouse models:

- HFUS visual grading [9,44,45]: the left lobe, anterior and posterior portions of the right lobe, left and right portions of the middle lobe, and the caudate lobe of the liver were imaged, and the following parameters were assessed:
  - Echostructure—score 1: homogenous liver parenchyma and regular hepatic surface; score 2 (mild steatosis): diffuse parenchymal mild heterogeneity, reduced visualization of the diaphragm and small peripheral vessels with no change on liver surface; score 3 (moderate steatosis): discrete coarse and heterogeneous parenchymal echogenicity, dotted or slightly irregular liver surface; score 4 (severe steatosis): extensive coarse and heterogeneous parenchymal echostructure, marked echogenicity, irregular or nodular hepatic surface with underlying regenerative nodules, obscured diaphragm and reduced visibility of kidney.
  - Echogenicity (relative to the renal cortex)—score 0: liver less echogenic than the renal cortex; score 1: hepatic echogenicity equal to the renal cortex; score 2: liver more echogenic than the renal cortex.
  - Presence of ascites—score 0: absent; score 1: present.
- Parametric analysis: Overall, normal hepatic parenchyma is less echogenic than the right renal cortex in rodents [7]. The hepatic echogenicity increases due to the presence of fatty infiltration and/or fibrosis, changing the relation between the liver and the right renal cortex [12].
  - Hepatic-renal ratio (HR): A longitudinal view was acquired in order to have both the liver (caudate lobe) and the right kidney clearly visualized. Liver echogenicity was compared with that of the renal parenchyma, to normalize differences in the overall ultrasound gain value used for the acquisitions. A region of interest (ROI, (0.1 ± 0.02 mm^2^) was manually drawn and placed in the liver parenchyma, avoiding focal hypo- and hyperechogenicity. A second ROI was positioned in correspondence with a portion of the renal cortex devoid of large vessels along the focusing area of the image at the same distance from the probe to avoid distorting effects in ultrasonic wave patterns. HR values were obtained by dividing the mean grey level of the hepatic ROI for that obtained for the renal one (pixel intensity = average intensity/mm^2^, arbitrary units, a.u.) [9,44].
  - Hepatic-portal vein ratio (HPV): Similarly, liver echogenicity was normalized to that corresponding to blood within the portal vein. Axial plane ultrasound images were acquired to visualize a portion of the portal vein in the center of the liver. One ROI (0.1 ± 0.02 mm^2^) was manually drawn and positioned within the lumen of the portal vein, while a second one was positioned in the liver parenchyma avoiding focal hypo- and hyperechogenicity, at the same depth and as close as possible to the center of the image, to maintain comparable ultrasound attenuation and avoid effects related to borderline echo distortion [9,44].
  - Gray-level histogram analysis of echogenicity (GLH): Liver images at different scanning planes (left lateral lobe, longitudinal; caudate lobe, longitudinal; right median lobe, axial) were analyzed using a gray-level histogram to obtain the quantitative mean and standard deviation values of echogenicity of each spatial region. Anatomical landmarks (greater curvature of stomach; cranial pole of the right kidney; porta hepatis, at the level which aorta, portal vein, and caudal vena cava are visible in cross-section) were chosen to scan reproducible imaging planes. ROIs (1 ± 0.02 mm^2^) were manually drawn in the liver parenchyma, avoiding focal hypo- and hyperechogenicity and as close as possible to the center of the image. This approach includes more representative parts of the liver parenchyma and avoids distortion of image artifacts, with good intra-observer reproducibility [46]. Changes in brightness and variance of the liver parenchyma were reported as follows: mean echogenicity of different lobes; standard deviation of brightness within ROI encompassing right median lobe as measure of tissue heterogeneity; standard deviation of brightness among ROIs in all planes imaged as measures of anisotropy [46,47].
  - Portal vein (PV) diameter: The evaluation of portal hypertension, through the measurement of the portal vein widening, is a recognized marker to non-invasively assess the severity of liver diseases [7,45].

Furthermore, renal alterations as a consequence of the WD have been investigated by HFUS. Since a good correlation of some ultrasonographic features with renal function has been demonstrated, cortical echogenicity and cortical thickness normalized for BW (CRT/BW) were calculated [48,49]. The renal cortical thickness was measured in the sagittal plane at the level of the mid-kidney over a medullary pyramid, perpendicular to the capsule, as was the distance from the corticomedullary junction to the renal capsule [48]. In addition, the renal pulsatility index (PI) and resistive index (RI) were measured. These parameters have been used in both clinical and animal research to assess pathological changes in renal hemodynamics related to the early stages of diabetic nephropathy or to systemic hemodynamic changes, such as *hr*, blood pressure, and cardiac output [50,51,52,53,54].

In particular, RI is considered an unbiased parameter for flow analysis, since it is the ratio of velocities. Normal RI value in adults would be in the range of 0.47–0.70, with differences of less than 5–8% between the two kidneys [55,56,57]. The RI value for 24-week-old C57Bl/6 male mice fed standard chow was approximately around 0.5 ± 0.15, while the PI value for the same mouse model at 21 weeks of age was 1.18 ± 0.19 (ultrasound examination performed under anesthetic regimen comparable to that used in our experiments as assumed hemodynamically neutral) [50,58]. In all 24-week-old mice, the right kidney was identified in the B-mode longitudinal plane with the same acquisition setting used for the liver, and the maximum longitudinal plane was evaluated, focusing on the renal papilla in the center of the organ. Color Doppler images of the renal vascular tree were used to identify an interlobar artery and guide the positioning of sample volume in correspondence of an intra-renal segmental artery. Blood flow was measured by pulse wave (PW) Doppler mode with a PRF (pulse repetition frequency) of 9–10 kHz. On the PW spectrum, the peak systolic velocity (PSV), the end diastolic velocity (EDV) and the velocity time integral (VTI) were calculated over an average of three to four cardiac cycles. Mean velocity (MV) was obtained by semi-automatically tracing the envelope of the flow signal corresponding to a single cardiac cycle. Based on these measurements, the RI was calculated as follows: RI = (PSV − EDV)/PSV [51,59], while the PI was assessed as (PSV − EDV)/MV [58]. After the imaging sessions, mice were allowed to recover completely in a heated cage, and monitored for signs of pain or discomfort.

### 2.5. Histological Examination

Liver was weighted, and samples of the organs of interest were formalin-fixed and embedded in paraffin as previously described [59]. Briefly, 7 μm sections were cut from each paraffin block and stained with hematoxylin and eosin (H&E) for morphological evaluation. Periodic acid–Schiff (PAS) and Masson’s trichrome staining were used to assess glycogen and collagen content, respectively. Two unblinded examiners, including an anatomical pathologist and a veterinarian with competencies in animal pathology (VB and SG) reviewed histological sections and performed the histopathological evaluation using a Nikon Eclipse E600 light microscope equipped with a digital camera. The morphometric analysis was performed using Nis element AR software version 3 (Nikon Instruments, Melville, NY, USA). Steatosis and inflammation were assessed on H&E-stained slides. At least five microscopic fields per section (400× magnification) and three section per animal were examined, including at least two different hepatic lobes; microscopic fields including hepatic venules and portal tracts were excluded. The pathological grades of fatty liver disease in each mouse were determined by the NAFLD preclinical scoring system [60], a validated histological score adapted in rodents from the human NASH–Cancer Research Network scoring system [61]. This simplified score comprises four histological features which are evaluated semi-quantitatively: steatosis: macrovescicular (score 0–3), microvescicular (score 0–3), hypertrophy (score 0–3); and inflammation (score 0–3). A global NAFLD activity score (NAS) was defined as the unweighted sum of the scores for steatosis, hypertrophy and inflammation, thus ranging from 0 to 9. In addition, the presence and extent of fibrosis was evaluated for NAFLD staging [61]. Changes from normal hepatocyte morphology were classified as borderline lesions according to steatosis, activity, fibrosis (SAF) scoring system [62], and differential diagnoses were made from glycogen accumulation [63]. Histological analysis of renal structural changes was performed on H&E- and PAS-stained sections on 20 glomeruli per mouse throughout the cortex, using a simplified scoring system according to that previously described in laboratory rodents [64]. In particular, the presence of morphological alterations of the glomerular areas was examined by two unblinded anatomists, with a semi-quantitative score as follows: score 0: none or <30% of glomeruli altered; score 1: >30–<70% of glomeruli altered; score 2: >70% of glomeruli altered.

### 2.6. Statistical Analysis

In this experimental protocol, we examined the effects of SD and WD in C57Bl/6J WT mice for future comparison with genetically modified mice, C57Bl/6J genetic background. The experimental group included 7–8 mice (n = 7–8). The sample size was calculated a priori, comparing means by extrapolating the expected data from relevant scientific literature [65] in order to obtain statistically significant results for differences of at least 30% (reference mean = 100; test mean = 70) between the averages of the measurements carried out for the various parameters examined (http://www.biomath.info/power/ttest.htm, accessed on 20 March 2021) [66]. A power of 80%, an expected variability of 20% (standard deviation = 20 March 2021) and a significance level of 5% (*p* ≤ 0.05) were considered. In evaluating the size of the experimental groups, consideration was given to the need to minimize the use of animals while maintaining sufficient power to detect significant effects. To account for expected attrition of animals throughout the duration of the study, the calculated sample size was adjusted by 10%. Further statistical analyses were performed using GraphPad Prism software version 8.0 (GraphPad Software, Inc., San Diego, CA, USA). Since the distribution of the data was unknown, variables were analyzed by the use of nonparametric tests where appropriate. The *t* test/Mann–Whitney test was used to compare differences in experimental outcomes between sex-and age- matched control and DIO groups. Repeated measures—a one-way ANOVA/Wilcoxon test was used to detect differences in experimental outcomes over time (8, 16, 24 weeks of age) in the same group. Repeated measures—a two-way ANOVA mixed effect model followed by Sidak’s multiple comparison post hoc test was used where appropriate, to compare group differences within time points of interest. Statistical significance was set at *p* < 0.05. Extreme values of the biochemical analysis of a 24-week-old male mouse in the SD group were excluded from the statistical calculations, and insulin sensitivity data were lost for one male mouse in WD group for insufficient sample quantity. Pearson correlations (one-tailed) were performed between the semi-quantitative and ordinal data of the NAS scoring system and the HFUS visual grading, as well as the numerical values of the parametric ultrasound analyses.

## 3. Results

### 3.1. WD Affects Body Weight and Nutritional Phenotype of C57Bl/6J Mice

**Figure 1:**
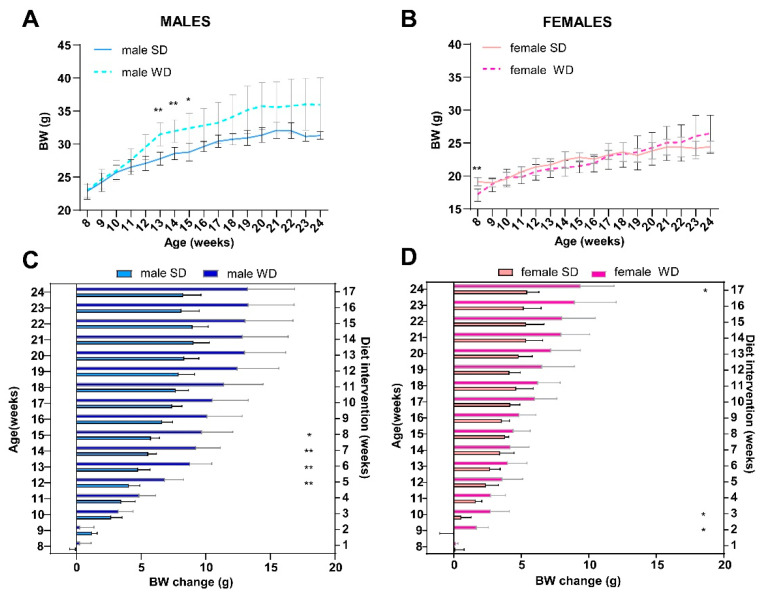
Effects of diet on BW. C57Bl/6J mice of WD groups had access to WD from 8 to 24 weeks of age. Body weight (g) in male (**A**) and female (**B**) mice were measured weekly. BW gain (g) relative to initial body weight value in male (**C**) and female (**D**) mice were calculated per experimental week. Values are reported as mean ± standard deviation. Data were compared using repeated measures—two-way ANOVA mixed effect model followed by Sidak’s multiple comparison post hoc test (* *p* < 0.05; ** *p* < 0.01).

As expected, BW and nutritional status of C57Bl/6J mice were significantly affected by WD. Baseline BW (g) was not different between male SD and WD groups (Mann–Whitney test, *p* = 0.7587), while it was statistically higher in the WD group between 13 and 15 weeks of age [ANOVA, diet: F (16, 208) = 5.978; *p* < 0.0001; Sidak’s test *p* < 0.05] (Figure 1A). Conversely, the female SD group was significantly heavier than the WD group at 8 weeks of age [Mann–Whitney test, *p* = 0.0005; ANOVA, diet: F (1, 14) = 0.08653; *p* < 0.7730; Sidak’s test *p* < 0.05] (Figure 1B), although all records over time were within the strain and age range provided by the supplier [32,67] and derived from the literature [68]. For that reason, BW gain (g) from baseline was correctly used for comparisons [69]. Consistently, WD increased rates of BW gain in both male and female mice (Figure 1C,D). In male mice, body weight increased significantly from 12 to 15 weeks of age [ANOVA, diet: F (1, 13) = 15.03, *p* < 0.0001; age: F (2.188, 28.45) = 121.2, *p* < 0.0001; age × diet: F (16, 208) = 7.182, *p* < 0.0001; Sidak’s test *p* < 0.01], while in female mice from 9 to 10 weeks of age and at the endpoint [ANOVA, diet: F (1, 14) = 11.43, *p* < 0.0001; age: F (3.235, 45.29) = 92.79, *p* < 0.0001; age × diet: F (16, 224) = 5.217 *p* < 0.0001; Sidak’s test *p* < 0.05].

**Figure 2:**
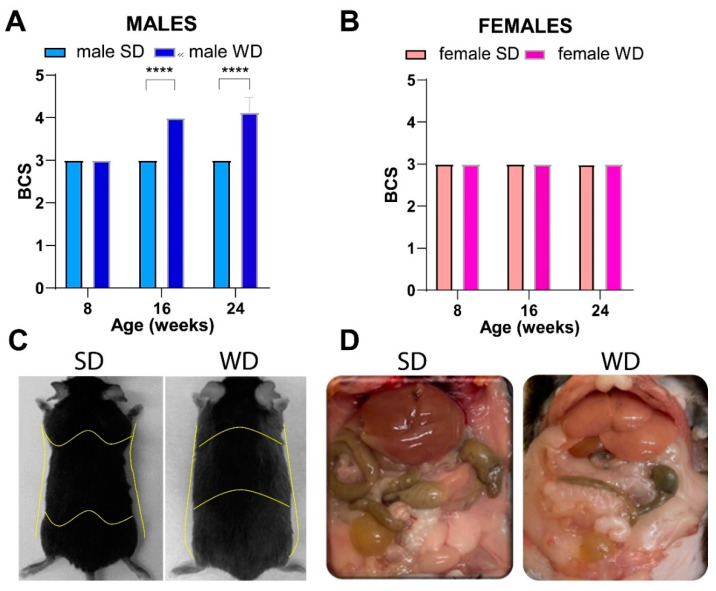
Effects of diet on body composition. BCS in male (**A**) and female (**B**) mice were measured every 8 weeks, starting from 8 weeks of age (onset of WD). Visual evidence of a relationship between BCS and body adiposity in SD- and WD-fed male mice at 24 weeks of age is shown in representative photographs: yellow lines highlight a rounded contour at the junction of the costal arch and upper part of the abdomen, scapular area, flank region, and lower abdomen profiles as anatomical landmarks likely due to enhanced subcutaneous and visceral white adipose tissue (**C**); intra-abdominal fat is increased, and liver shows a macroscopically pale and “fatty” appearance (**D**) in response to WD. Values are reported as mean ± standard deviation. Data were compared using repeated measures two-way ANOVA mixed effect model followed by Sidak’s multiple comparison post hoc test (**** *p* < 0.0001).

Differently, the WD intervention produced a significant increase in BCS only in C57BL/6J males at 16 and 24 weeks of age [ANOVA, diet: F (1, 14) = 289.0; age: F (1.000, 14.00) = 73.00; age × diet: F (2, 28) = 73.00; *p* = 0.0001; Sidak’s test *p* < 0.05] (Figure 2A,B). This finding was consistent with visual evidence of increased subcutaneous, abdominal, and gonadal adipose tissue depots, as well as “fatty liver” appearance at necropsy of male mice [20], suggesting sex-dependent differences in fat distribution patterns (Figure 2C,D).

**Figure 3:**
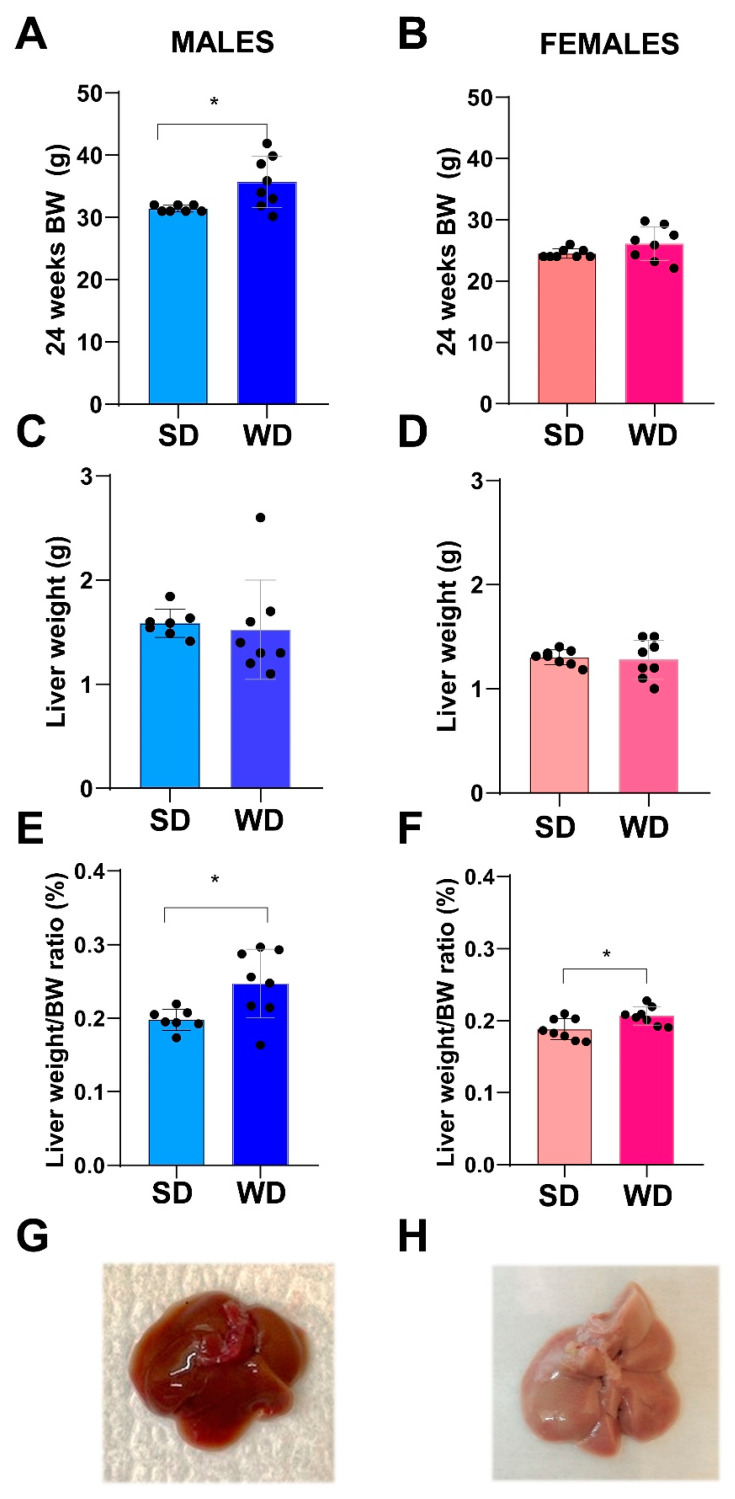
Effects of diet on liver. Comparisons between livers from SD- and WD-fed mice. Final BW (g) (**A**,**B**), liver weight (g) (**C**,**D**), and liver weight/BW ratio (%) (**E**,**F**) were calculated at 24 weeks of age. Values are mean ± standard deviation. Data were compared using Mann–Whitney test (* *p* < 0.05). Macroscopic appearance of the liver at autopsy: representative photos of the excised liver in a male SD-fed and a WD-fed mouse (**G**,**H**), respectively: liver shows a pale color and rounded margins in response to WD.

At 24 weeks of age, the BW (g) differed significantly only between male groups (Mann–Whitney test, *p* = 0.0342) (Figure 3A,B). Overall, liver weight (g) was not significantly different between SD- and WD-fed groups (Figure 3C,D), while the ratio of liver weight to body weight (%) (Figure 3E,F) was significantly higher in the WD-fed males and females compared to the control groups (Mann–Whitney test, *p* = 0.0289 and *p* = 0.0379, respectively).

### 3.2. WD Influences Feeding Behavior in C57Bl/6J Substrain

**Figure 4:**
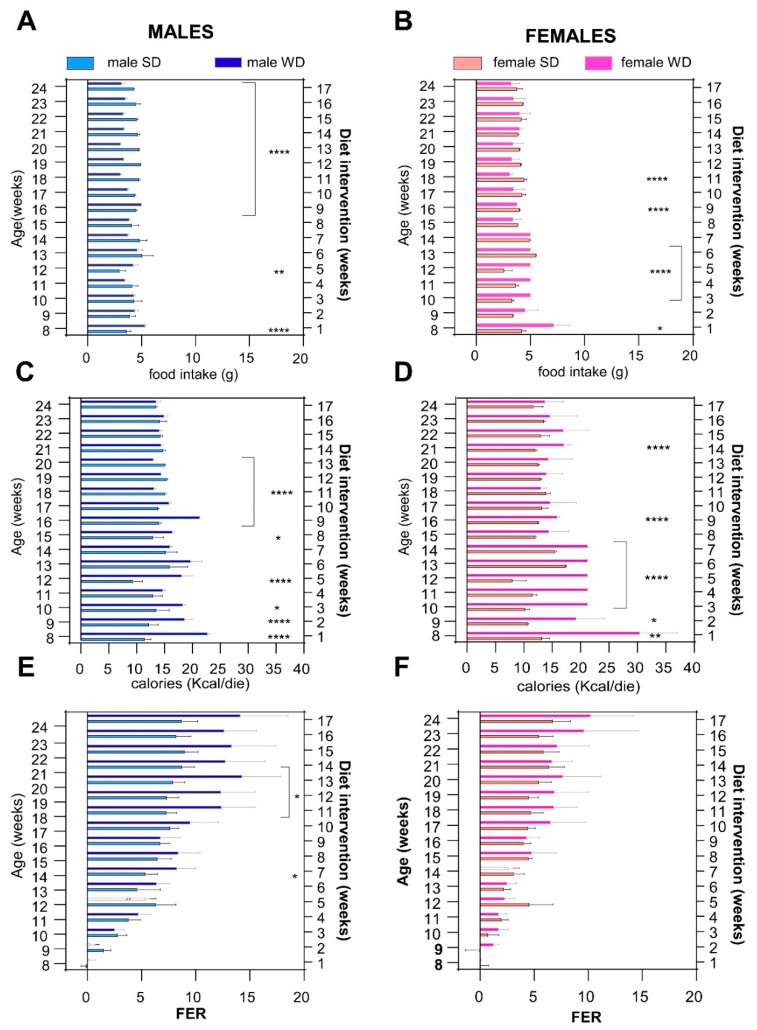
Effects of diet on feeding behavior. Daily food intake (g) (**A**,**B**), caloric intake (kcal) (**C**,**D**) and FER [weight gained (g)/kcal consumed (kcal)] (**E**,**F**) were calculated for each experimental week. Data were compared using repeated measures—two-way ANOVA mixed effect model followed by Sidak’s multiple comparison post hoc test (* *p* < 0.05; ** *p* < 0.01; **** *p* < 0.0001).

Overall, WD influenced food intake in an age- and sex-specific manner (Figure 4). The male WD group had significantly higher food intake at 8 and 12 weeks, while eating significantly less from 16 to 24 weeks of age than SD males (ANOVA, diet: F (1, 13) = 132.5, *p* < 0.0001; age: F (1.826, 23.73) = 15.27, *p* < 0.0001; age × diet: F (16, 208) = 35.31, *p* < 0.0001; Sidak’s test *p* < 0.0001). Female WD mice ate significantly more than the sex-matched control group from 8 to 12 weeks of age, while eating significantly less at 16 and 18 weeks of age [ANOVA, diet: F (1, 14) = 1.754; *p* < 0.2066; age F (1.348, 18.87) = 24.31; *p* < 0.0001; age × diet F (16, 224) = 24.31, *p* < 0.0001; Sidak’s test *p* < 0.0001], with a pattern similar to what was described in males (Figure 4A,B).

Assuming that WD had a higher caloric density than SD, male WD group showed higher calorie intake than SD group from 8 to 17 weeks of age, while it was lower thereafter, and nearly isocaloric from 21 to 24 weeks of age, reaching statistical significance at different time points [ANOVA, diet: F (1, 13) = 279.6, *p* < 0.0001; age: F (2.047, 26.62) = 22.18, *p* < 0.0001; age × diet: F (16, 208) = 46.17, *p* < 0.0001; Sidak’s test *p* < 0.05–*p* < 0.0001]. The female WD group showed a significantly higher caloric intake than the female SD group from 8 to 16 weeks of age but, unlike males, the trend never reversed and the isocaloric phase was reached at 22 weeks of age [ANOVA, diet: F (1, 14) = 70.44, *p* < 0.0001; age × diet: F (16, 224) = 26.79, *p* < 0.0001; Sidak’s test *p* < 0.05–*p* < 0.0001] (Figure 4C,D).

The male WD group displayed a greater FER than the male SD group from 13 weeks of age, reaching significance at 14 and from 18 to 20 weeks of age [ANOVA, diet: F (1, 13) = 10.27, *p* < 0.0001; age: F (2.013, 26.16) = 96.11, *p* < 0.0001; age × diets: F (16, 208) = 10.67, *p* < 0.0001; Sidak’s test *p* < 0.05]. Conversely, FER was not significantly different between female WD mice and the sex-matched control group at any time point [ANOVA, age: F (2.276, 31.86) = 60.16; *p* < 0.0001; age × diet: F (2.276, 31.86) = 60.16; *p* < 0.0001] (Figure 4E,F). Taken together, these results suggest that the trend for greater BW gain in WD-fed C57Bl/6J might be explained by a temporary hyperphagic phase observed within the first 10 week of diet, followed by a normalization phase. However, FER was significantly different only between male WD- and SD-fed mice in the last experimental weeks, when WD-fed mice developed an overweight phenotype, even though they ate less and consumed an equivalent amount of calories to SD-fed mice.

### 3.3. WD Induces Changes in Lipid Metabolism of C57Bl/6J Substrain

**Figure 5:**
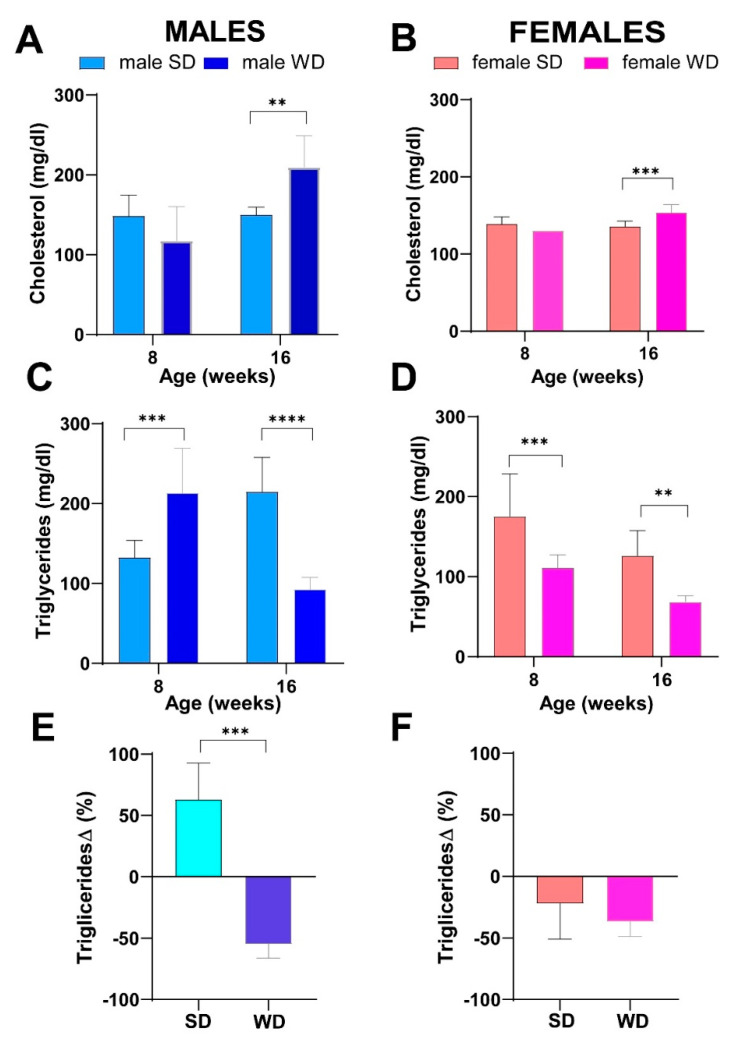
Effects of diet on lipid homeostasis. Non-fasting total cholesterol (mg/dL) (**A**,**B**) and non-fasting triglycerides (mg/dL) (**C**,**D**) at 8 and 16 weeks of age in male and female mice, respectively. Data were compared using repeated measures—two-way ANOVA mixed effect model followed by Sidak’s multiple comparison post hoc test. Changes in non-fasting triglycerides from baseline (%) (**E**,**F**) were evaluated by Mann–Whitney test in male and female mice, respectively (** *p* < 0.01; *** *p* < 0.001; **** *p* < 0.0001).

Non-fasting basal total cholesterol levels were not significantly different between male groups, while, reasonably, WD-fed mice developed hypercholesterolemia at 16 weeks of age [ANOVA, age: F (1, 13) = 11.28; *p* = 0.0051; age × diet: F (1, 13) = 10.67, *p* = 0.0061; Sidak’s test *p* > 0.05] (Figure 5A). Conversely, male WD group had significantly higher non-fasting triglycerides at 8 weeks of age, whereas the trend was reversed at 16 weeks compared to the SD group [ANOVA, age × diet: F (1, 13) = 10.67, *p* = 0.0061; Sidak’s test *p* > 0.01] (Figure 5C).

For that reason, changes in plasma triglycerides from baseline were used for comparisons, and the significance of triglycerides decrease at 16 weeks of age was confirmed in the WD group (Mann–Whitney test *p* = 0.0003) (Figure 5E).

Superimposable results were observed between female groups for non-fasting total cholesterol [ANOVA, age: F (1, 14) = 15.03; *p* = 0.0017; age × diet: F (1, 14) = 25.66, *p* = 0.0002; Sidak’s test *p* > 0.001] (Figure 5B). Furthermore, the female SD group also showed significantly higher values of non-fasting triglycerides at 8 weeks, as well as at 16 weeks of age [ANOVA, diet: F (1, 14) = 29.23; *p* < 0.0001; age: F (1, 14) = 23.89; *p* = 0.0002; Sidak’s test *p* < 0.05] (Figure 5D), but these results were not confirmed comparing changes in plasma triglycerides from baseline (Figure 5F).

**Figure 6:**
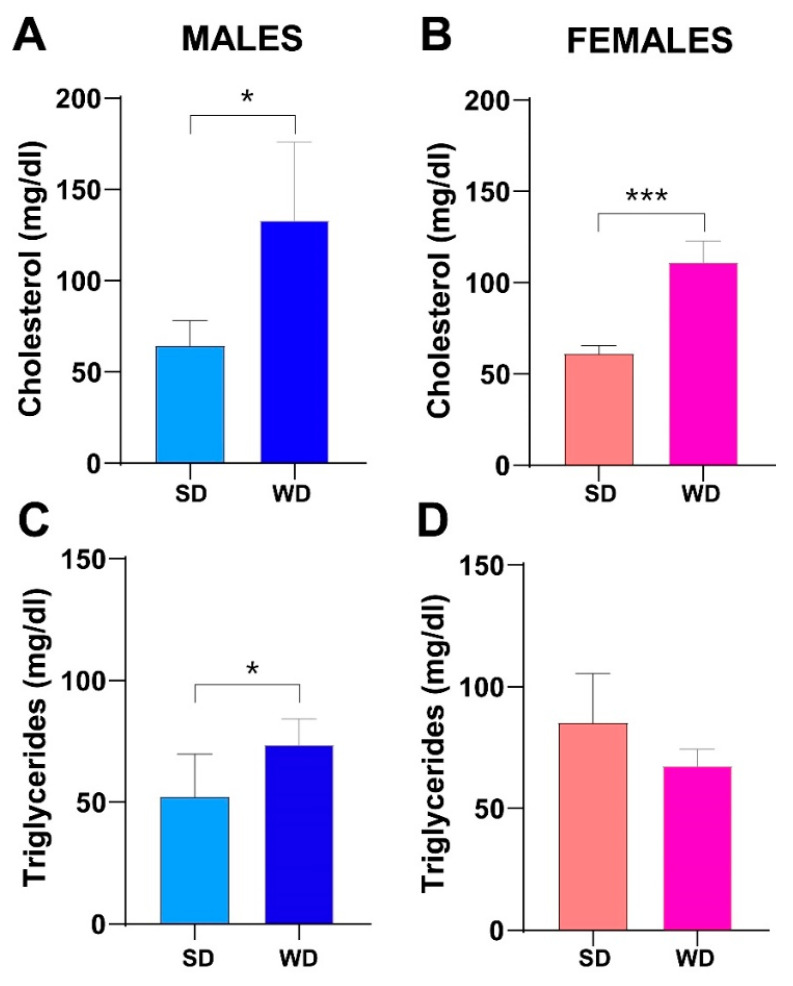
Effects of diet on lipid homeostasis. Fasting total cholesterol (mg/dL) (**A**,**B**) and fasting triglycerides (mg/dL) (**C**,**D**) at 24 weeks of age in male and female mice, respectively. Data were compared using Mann–Whitney test between sex-matched groups (* *p* < 0.05; *** *p* < 0.001).

Consistently, fasting total cholesterol was also significantly higher in the WD group of both sexes compared to SD-fed counterparts at 24 weeks of age (Mann–Whitney test *p* = 0.0143 and *p* = 0.0002, respectively) (Figure 6A,B). Conversely, at the same age, fasting triglycerides were significantly greater only in male WD group compared to SD-fed controls (Mann–Whitney test *p* = 0.0370) (Figure 6C,D).

Considering the above results, the diet-induced BW gain and changes in macroscopic liver appearance were overall associated with progressive hypercholesterolemia in both female and male WD-fed mice. Unexpectedly, non-fasting triglycerides showed a significant reduction at 16 weeks of age only in WD-fed males. This finding has been described in other studies on C57Bl/6J mice fed a high-fat, high-cholesterol diet [11,70]. The hypothesis was that this specific diet might reduce synthesis and/or increase clearance of triglycerides or decrease hepatic VLDL secretion and/or increase absorption of triglycerides from adipose tissue, due to the acute increase in insulin [11]. Furthermore, the expression of lipoprotein lipase might be induced in the liver of adult mice with high dietary cholesterol, lowering plasma triglycerides levels [71]. Finally, these results could be interpreted considering the known sex- and age-related differences in serum biochemical parameters of C57BL/6J mice [30] and also based on the vending source of mouse substrain [72,73].

### 3.4. WD Impairs Glucose Homeostasis in C57Bl/6J Substrain

**Figure 7:**
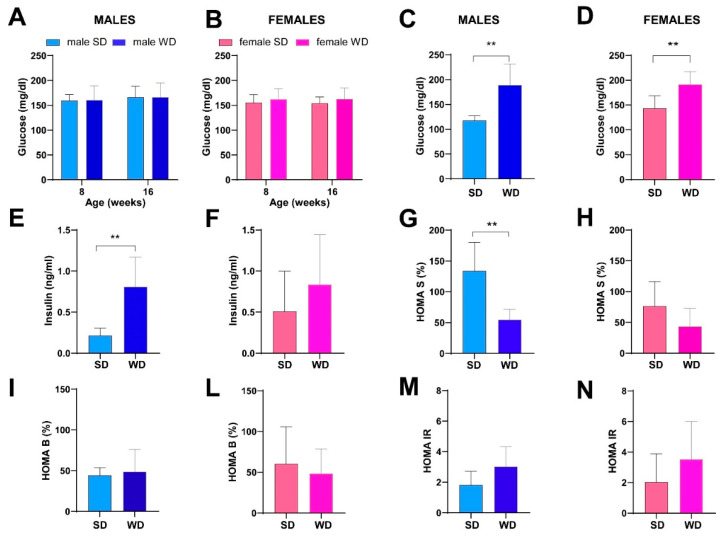
Effects of diet on glucose homeostasis and insulin sensitivity. Non-fasting glucose (mg/dL) (**A**,**B**) at 8 and 16 weeks of age in male and female mice, respectively. Data were compared using repeated measures—two-way ANOVA mixed effect model followed by Sidak’s multiple comparison post hoc test. Fasting glucose (mg/dL) (**C**,**D**), fasting insulin (ng/mL) (**E**,**F**), HOMA S (%) (**G**,**H**), HOMA B (%) (**I**,**L**) and HOMA IR (**M**,**N**) indexes at 24 weeks of age in male and female mice. Data were compared using the Mann–Whitney test between sex-matched groups (** *p* < 0.01).

No significant changes over time in non-fasting blood glucose (mg/dL) were observed between sex-matched SD- and WD-fed groups (Figure 7A,B). However, two males in the WD-fed group showed mild hyperglycemia at 16 weeks of age. At 24 weeks of age, fasting blood glucose was significantly higher in the WD group of both sexes compared to the SD-fed counterparts (Mann–Whitney test, males: *p* = 0.0002 and females: *p* = 0.0030) (Figure 7C,D). Furthermore, the male WD group showed a significantly higher fasting insulin and lower HOMA-%S (Mann–Whitney test *p* = 0.0012 and *p* = 0.0043, respectively) (Figure 7E,G). In the male WD group, a trend towards impaired β-cell function (HOMA-%B) and insulin resistance (HOMA-IR) was observed, although it was not statistically significant (Figure 7I,M), similarly to the female WD group (Figure 7F,H,L,N).

These findings are consistent with the sexual dimorphism in glucose homeostasis described in the C57BL/6J mouse strain, although few studies have evaluated the metabolic effects of WD in female mice [1]. Furthermore, the higher insulin levels and lower insulin sensitivity found in WD-fed male mice are concordant with the obvious overweight phenotype and fatty liver appearance, as well as clear dyslipidemia, at 24 weeks of age.

### 3.5. WD Induces Changes in Hepatic and Renal Biochemistry of C57Bl/6J Mice

**Figure 8:**
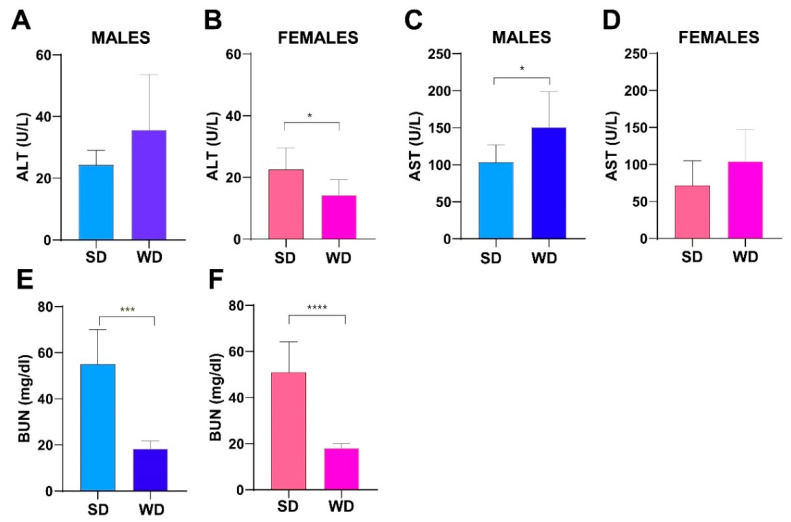
Effects of diet on liver and kidney function. ALT (U/L) and AST (U/L) values (**A**–**D**) and BUN (mg/dL) (**E**,**F**) at 24 weeks of age in male and female mice. Data were compared using Mann–Whitney test between sex-matched groups (* *p* < 0.05; *** *p* < 0.001; **** *p* < 0.0001).

The male WD group showed higher ALT (U/L) and AST (U/L) values compared to SD-fed counterpart, reaching statistical significance for AST (Mann–Whitney test *p* = 0.002) (Figure 8A,C). A similar trend was observed between SD-fed and WD-fed female groups for AST. Conversely, female WD group showed significantly lower ALT values compared to sex-matched controls (Mann–Whitney test *p* = 0.0134) (Figure 8B,D). However, liver enzymes were in the normal range reported in the literature for both SD-fed and WD-fed mice [73,74]. Interestingly, BUN (mg/dL) was significantly higher in the SD groups of both sexes (Mann–Whitney test, males: *p* = 0.0003 and females: *p* = 0.0001) (Figure 8E,F), although it was in the reference range for healthy mice. Taken together, the changes observed in transaminases and azotemia would suggest a mild effect of WD on the metabolism of various organs, such as liver, heart, muscles, pancreas and kidneys [75], and related to decreased conversion of ammonia to urea in hepatic diseases and/or to low-protein diet [76]. Furthermore, increased visceral fat and decreased muscle mass in metabolic syndrome are gaining attention as risk factors for fatty liver disease and have been found to be related to decreased BUN value [77].

### 3.6. WD Causes Progressive Structural and Functional Changes in the Heart, Liver, and Kidney of C57Bl/6J Mice That Can Be Detected Early and Monitored In Vivo by HFUS

All animals showed normal sinus rhythm on ECG, and based on the visual wall motion assessed, no evident myocardial contraction abnormalities of the left ventricle were captured on the B-mode movie frames acquired in the long and short axis views.

Descriptive statistics (mean ± SD) for echocardiographic morphometric and functional parameters over time are reported in Table 1. ANOVA for repeated measures was used to evaluate the differences between the sex-matched diet groups concerning the echocardiographic parameters. Since post hoc test revealed that some normalized echocardiographic measurements at 8 weeks of age were statistically different between the sex-matched groups, the magnitude of changes from baseline was used in these cases for comparisons. In male mice, IVS/LVAW; s variations over time showed a significative interaction between age and diets [ANOVA, F (1, 13) = 8.393, *p* = 0.0125]. LVPW; d values were significantly lower in WD-fed males at 16 and 24 weeks compared to SD-fed counterpart [ANOVA, diet: F (1, 13) = 15.61, *p* = 0.0017; age: F (1.585, 20.61) = 4.283, *p* = 0.0354; Sidak’s test *p* < 0.05], as well as LVPW; s values [ANOVA, diet: F (1, 13) = 6.011, *p* = 0.0291; age: F (1.946, 25.30) = 5.476, *p* = 0.0111]. Global systolic function parameters EF and FS showed no statistical differences between SD- and WD-fed male groups, as well as LV mass corrected, diastolic and systolic LV vol; d, LV vol; s, hr, SV and CO. Although the post hoc test showed no statistical differences between SD- and WD-fed male mice at any time point, RWT was significantly affected by age and diet, with a higher tendency for SD-male mice [ANOVA, diet: F (1, 13) = 10.35, *p* = 0.0067; age: F (1.833, 23.83) = 11,38, *p* = 0.0005]. In female mice, EF, FS, LV mass-corrected and hr showed no statistical differences between SD- and WD-fed female groups. In contrast to males, SV and CO showed significant changes with age as well as a significant interaction between age and diets, showing a lower trend in female WD-fed mice [ANOVA, diet: CO: F (1, 14) = 6.523, *p* < 0.0229; age, SV, F (1.422, 19.91) = 12,17, *p* = 0.0010—CO, F (1.405, 19.67) = 7.852, *p* = 0.0063; age × diets, SV: F (2, 28) = 9.257, *p* = 0.0008—CO, F (2, 28) = 5.614, *p* = 0.0089; Sidak test CO 16 weeks of age, *p* = 0.0074]. Finally, the magnitude of RWT changes in female mice was significantly affected by age and the interaction between age and diets, with a higher trend for WD-fed mice [ANOVA, age: F (1, 14) = 6.749, *p* = 0.0211; age × diets: F (1, 14) = 4.618, *p* = 0.0496; Sidak test at 16 weeks of age, *p* = 0.0175].

**Figure 9:**
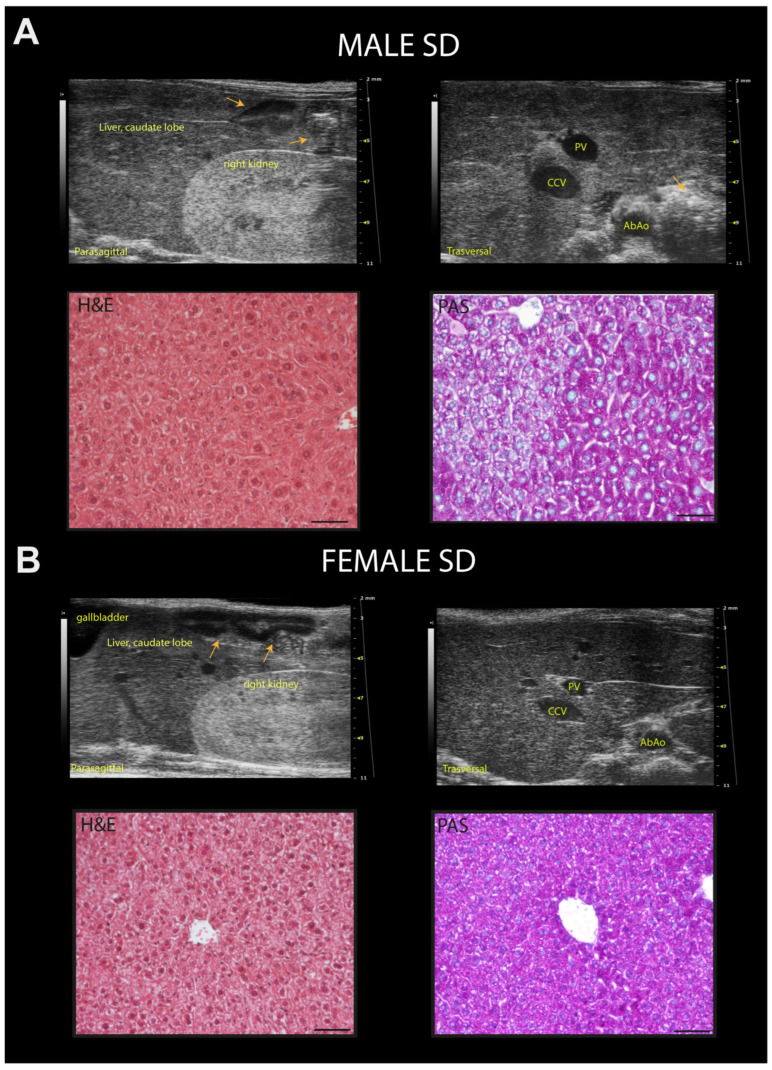
Representative ultrasound images and correlation with histology of C57Bl/6J mice fed a standard diet. (**A**) Example of normal rodent liver/kidney echogenicity ratio in a 24-week-old male mouse, with less echogenic hepatic parenchyma than the right renal cortex, comparable to that observed at 8 and 16 weeks of age (upper row, left); homogenous hepatic parenchymal echogenicity, regular liver surface, sharp margins, distinct visualization of the portal vein vessels, with finely coarsened echotexture (upper row, right). Histological evaluation of the same hepatic parenchyma in HFUS images showing hepatocytes with a pale cytoplasm and hepatocytes with more eosinophilic cytoplasm (H&E); PAS staining confirmed physiologic cytoplasmic glycogen collections. (**B**) Example of normal, liver/kidney echogenicity ratio, constant over time in a 24-week-old female mouse (upper row, left); homogenous liver parenchymal echogenicity and echostructure, regular hepatic surface, sharp margins, distinct visualization of the portal vein vessels (upper row, right). Histological evaluation of the same liver parenchyma in US images showing homogenous distribution of hepatocytes with eosinophilic cytoplasm (H&E); PAS staining highlights the presence of diffuse physiologic cytoplasmic glycogen (lower row, right). Scale bar 50 µm. Orange arrows: intestinal loops with mucous or gaseous pattern, associated with shadow effects; PV: portal vein; CCv: caudal vena cava; AbAo: abdominal aorta.

**Figure 10:**
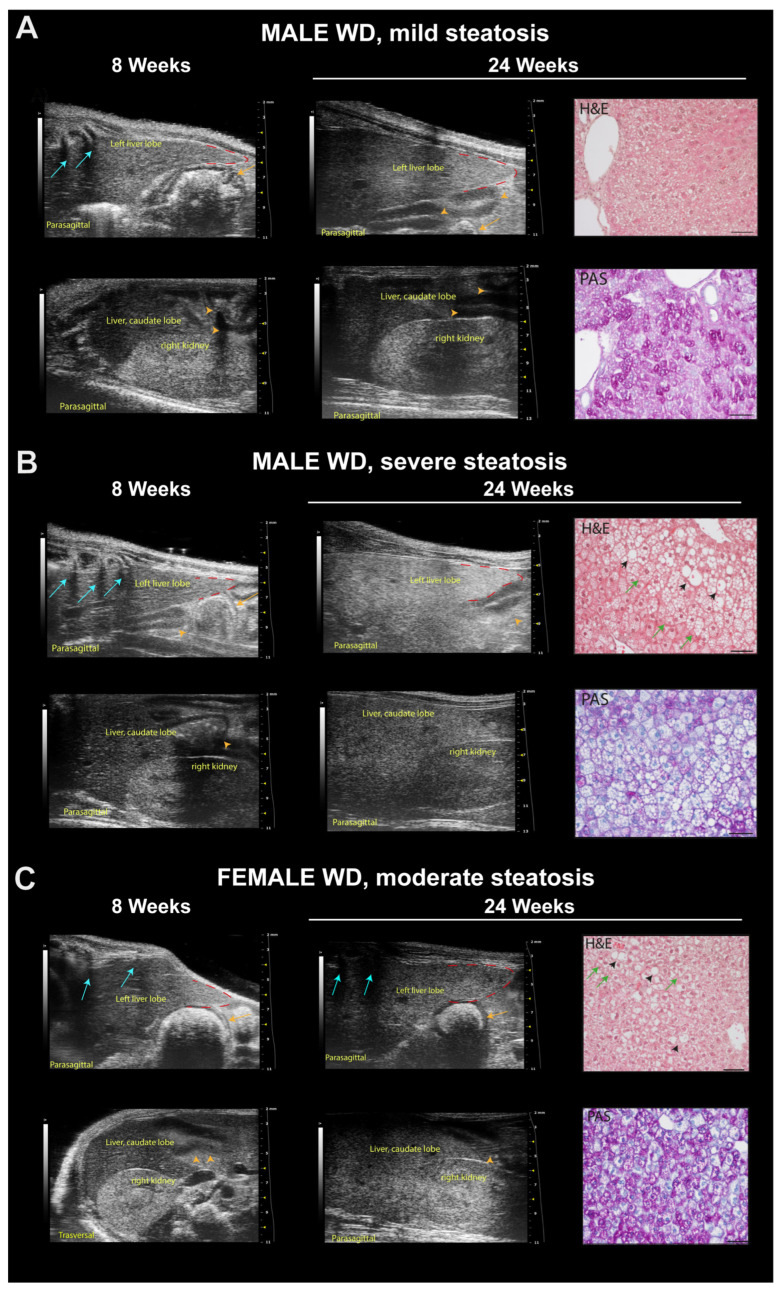
Representative ultrasound images and correlation with histology of C57Bl/6J mice fed a Western diet. (**A**) Example of mild steatosis in a 24-week-old male mouse, with liver parenchyma even less echogenic than the right renal cortex (score 0), but diffusely increased parenchymal echogenicity and slightly rounded lobe edges (score 2) compared to those observed at 8 weeks of age. H&E histological evaluation of the same liver parenchyma showed “patches” of round-shaped hepatocytes with pale, reticulated cytoplasm, of normal size or enlarged some of which corresponded to physiological deposits of glycogen in the cytoplasm (PAS). Scale bar 50 µm. (**B**) Example of severe steatosis in a 24-week-old male mouse, showing a marked increase in echogenicity, reduced visibility of portal vessels, enlarged volume of the liver extending caudally to the costal arch, rounded margins (red dotted line), coarse and heterogeneous parenchymal echostructure (score 4); caudate hepatic lobe echogenicity was greater than the right renal cortex echogenicity (score 2) compared to those observed at 8 weeks of age. H&E histological of the same liver parenchyma showed diffuse fatty liver, with large lipid droplets (black arrowheads), swollen hepatocytes containing small lipid droplets (microvesicular steatosis, green arrows). PAS staining confirmed the presence of lipid accumulation showing large white areas. Scale bar 50 µm. (**C**) Example of moderate steatosis in a 24-week-old female mouse, displaying discrete coarse and heterogeneous parenchymal echogenicity (score 3) and hepatic echogenicity equal to the renal cortex (score 1) compared to those observed at 8 weeks of age. H&E histological of the same liver parenchyma showed diffuse microvesicular steatosis (green arrows) evolving into large lipid droplets (black arrowheads). PAS staining confirmed the presence of hepatocytes with microvesicular steatosis (white areas) and with glycogen deposits (pink areas). Scale bar 50 µm. Orange arrows: stomach; orange arrowheads: intestinal loops with mucous or gaseous pattern, associated with shadow effects; blue arrows: acoustic shadowing by the ribs.

Qualitative ultrasound examination of liver at baseline showed no altered echogenicity and echostructure in all mice. Visual inspection highlighted that at 24 weeks of age, 14% of SD-fed male mice showed a slightly diffuse increase in liver echogenicity/heterogeneity of echotexture and, in all mice, liver was less echogenic than the renal cortex. Conversely, WD-fed male mice showed a diffusely increased echogenicity (12.5%), a discretely (16.6%) or extensively coarsened and heterogeneous parenchyma (12.5%), whose brightness was higher to the right renal cortex in some mice (25%). At the same age, all SD-fed female mice showed no obvious ultrasonographic alterations of the liver, while WD-fed female mice showed a diffusely increased parenchymal echogenicity (62.5%) or discretely coarsened and heterogeneous parenchyma (37.5%), as well as liver brightness nearly equal to the right renal cortex in some mice (25%). The results of the qualitative ultrasound examination of liver were in agreement with histological findings (see Table 2, Figure 9 and Figure 10, and Section 3.7 for details).

In multiparametric analysis, all ultrasound measures analyzed at baseline were not statistically different between the sex-matched groups.

**Figure 11:**
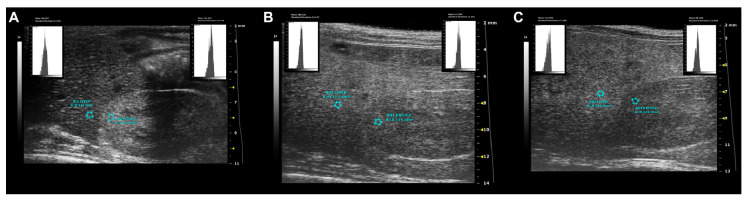
Standardized ultrasound hepatic/renal ratio, representative ultrasound parametric imaging for longitudinal assessment of fatty liver progression: (**A**) 8-week-old C57Bl/6J male mouse, baseline examination prior to Western-type diet initiation: normal liver/kidney ratio in rodents, with hepatic parenchyma less echogenic than the right renal cortex (HR = 0.79); (**B**) the same mouse shows hepatic echogenicity equal to the renal cortex at 16 weeks of age (HR = 1.01) and (**C**) increased liver echogenicity compared to right renal cortex at 24 weeks of age (HR = 1.25). The graphic representation of the region of interest (ROI) circles shows the gray scale distribution (signal brightness intensity expressed in arbitrary units, a.u.; blue bar = mean value; green bars = standard deviation) of the pixels in the selected liver, right kidney cortex.

WD-fed female group showed a progressive increase in HR index from 16 to 24 weeks of age compared to SD-fed female group [ANOVA, diet: F (1, 14) = 29.76, *p* < 0.0001; age: F (1.649, 23.09) = 6.939, *p* = 0.0065; age × diet: F (2, 28) = 0.3446, *p* = 0.0459; Sidak’s test, females: 16 weeks, *p* = 0.0005; 24 weeks, *p* = 0.0033] (Figure 11).

A similar trend was observed in the WD-fed male group compared to the SD-fed counterpart at the same time points, although no significance was reached.

Comparable results were obtained between female groups regarding HPV ratio [ANOVA, diet: F (1, 13) = 12.50, *p* < 0.0037; age: F (1.576, 20.48) = 9767, *p* = 0.0019)].

**Figure 12:**
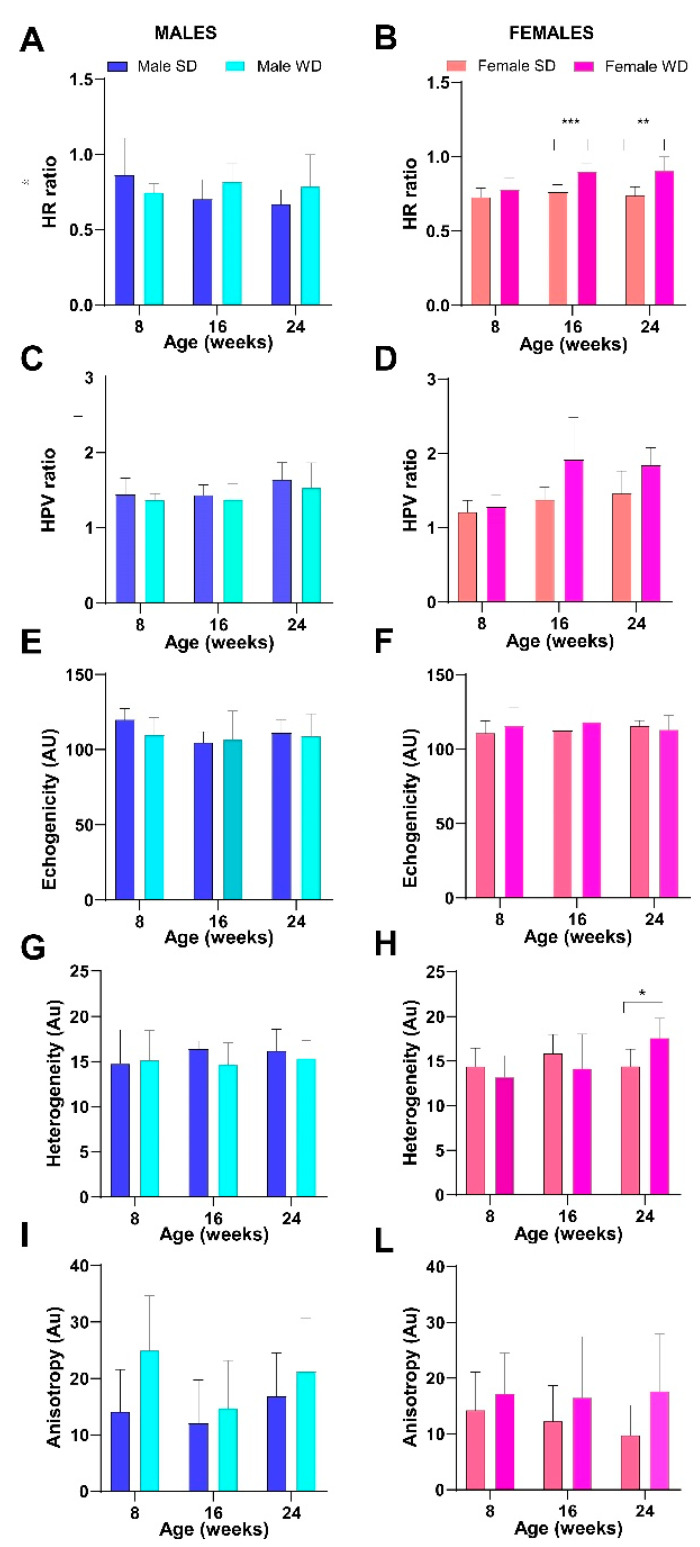
Effects of diet on liver echogenicity. HFUS measures of tissue brightness intensity (**A**–**F**) and tissue brightness variance (**G**–**I**,**L**). Data were compared using repeated measures two-way ANOVA mixed effect model followed by Sidak’s multiple comparison post hoc test between sex-matched groups (* *p* < 0.05; ** *p* < 0.01; *** *p* < 0.001).

As a representation of the local variation in tissue echogenicity, heterogeneity of right median liver lobe showed significant effects of interaction between diet intervention and age in females at 24 weeks of age [ANOVA, age × diet F (2, 28) = 4.434, *p* = 0.0212; 24 weeks Sidak’s test *p* = 0.0258]. Anisotropy, as a measure of regional variation in tissue brightness across different lobes, showed significant changes across different diets in mice of both sexes, with WD-fed mice showing a trend toward higher values than age- and sex-matched SD-fed mice [ANOVA, diet: males, F (1, 13) = 6.430, *p* = 0.0249—females, F (1, 14) = 7.899, *p* = 0.0139] (Figure 12).

**Figure 13:**
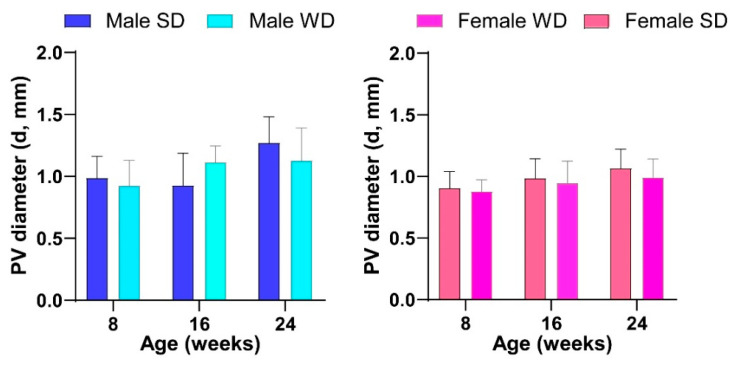
Effects of diet on liver vessels. HFUS measures of portal vein diameter. Data were compared using repeated measures two-way ANOVA mixed effect model followed by Sidak’s multiple comparison post hoc test.

Finally, the pattern of temporal changes in PV diameter differed between SD- and WD-fed male mice, although this value was not statistically different at any time point in both sex-matched comparisons [ANOVA, age: males, F (1.965, 25.54) = 8.409, *p* = 0.0016—females, F (1.542, 21,58) = 3.617, *p* = 0.0544; age × diets: males, F (2, 26) = 3.894, *p* = 0.0332] (Figure 13).

**Figure 14:**
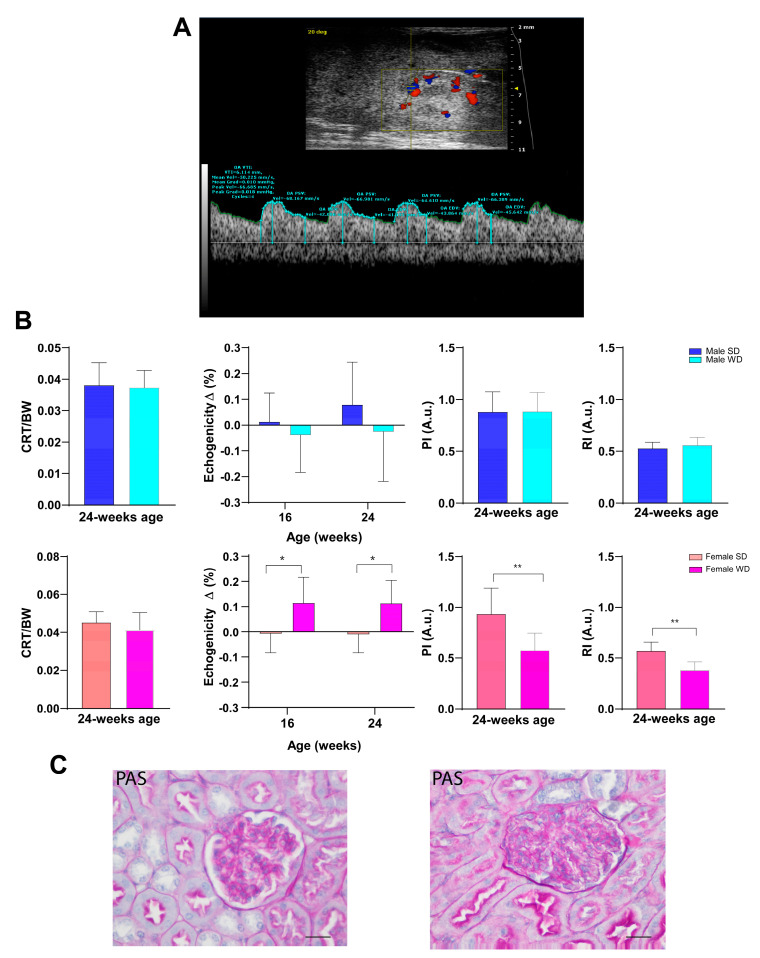
(**A**): Representative ultrasound image of kidney performed using B-mode for measurement of intra-renal flow from a male C57Bl/6J fed a Western-type diet. Color Doppler overlay was used to locate the intra-renal arteries. Pulse wave Doppler mode shows the typical peak systolic velocity (PSV) and the end-diastolic velocity (EDV) in renal arteries. (**B**): Morphofunctional analysis of diet effects on kidney. CRT/BW at 24 weeks of age and changes in tissue brightness intensity over time were measured. PI and RI were calculated from the spectral data. Data were compared using repeated measures two-way ANOVA mixed effect model followed by Sidak’s multiple comparison post hoc test or Mann–Whitney test between sex-matched groups. (**C**): Representative histological images of kidney in 24-week-old female mice (PAS, 400×, scale bar 20 µm), showing a normal glomerulus in the SD-fed group (left) and altered glomerulus in the WD-fed group (right) (* *p* < 0.05; ** *p* < 0.01).

Over time, all animals showed a normal ultrasound appearance of the right kidney, characterized by the thin, bright line of the renal capsule, a regular and defined renal cortical margin, the medulla in the medial portion of the kidney darker than the surrounding cortex, and the pelvis almost entirely occupied by the papilla [53]. Although WD-fed mice showed slightly lower normalized cortical thickness values, no significant differences were found in CRT/BW between sex-matched groups fed different diets. However, unlike males, ANOVA showed a significant increase in renal cortex echogenicity from baseline in WD-fed female mice compared to SD-fed ones [ANOVA, diet: F (1, 14) = 8.796, *p* = 0.0102]. At 24 weeks of age, PI and RI were statistically different between SD- and WD-fed female mice (Mann–Whitney test, *p* ≤ 0.01) and compared to the reference range. This finding could be likely related to the lower trend for SV and CO found in WD-fed females compared to SD-fed ones [51] (Figure 14).

### 3.7. WD Induces Histological Changes in the Liver and Kidney of C57Bl/6J Mice

The pathological grades of fatty liver disease in each mouse were determined by the NAFLD preclinical scoring system as reported in the Material and Methods section. Briefly, in microvesicular steatosis, small, clearly defined lipid droplets are widespread in the cytoplasm of hepatocytes, whereas large lipid droplets that occupy almost the entire cytoplasm of hepatocytes characterize macrovesicular steatosis. Lipid droplets appear optically empty in H&E- and PAS-stained sections and progressively displace cell nuclei marginally. Hypertrophic hepatocytes have the same cytoplasmic characteristics but appear much larger than surrounding ones. In Table 3, the total score assigned for the most relevant histological features in murine liver samples is listed by grading category and the number of subjects per score is shown.

In H&E-stained liver sections from 24-week-old mice fed SD of either sex, hepatocytes showed a normal appearance with abundant cytoplasm and centrally located round nuclei. The PAS staining revealed glycogen-rich areas, allowing the differential diagnosis of physiological glycogen accumulation in mouse liver from borderline pathological foam cells [78]. In WD-fed female mice, 37.5% of subjects showed the lowest grade of macrovesicular hepatic steatosis, while 75% displayed the lowest grade of microvesicular hepatic steatosis and/or hypertrophic hepatocytes encompassing the entire grading window. Mild liver inflammation was observed in one (12.5%) and mild fibrosis in two (25%) WD-fed female mice. Three WD-fed male mice (37.5%) showed histological features of borderline “ballooning” lesions, characterized by “patches” of hepatocytes with round shape and pale, reticulated cytoplasm, with normal or enlarged size, as confirmed by a comparison of serial sections stained with H&E and PAS. Macrovesicular hepatic steatosis at the lowest grade was observed in 62.5% of WD-fed male mice, in almost all cases (50%) associated with microvesicular hepatic steatosis in a mixed pattern. No hypertrophic hepatocytes and inflammation were observed, while mild fibrosis was found in the liver of 50% of WD-fed males. A moderate positive linear correlation was found in male WD mice between US visual grading and NAFLD histological score (Pearson r = 0.6379; *p* = 0.0444), HR index (Pearson r = 0.6604; *p* = 0.0373) and mean liver echogenicity (Pearson r = 0.7214; *p* = 0.0217). Examination of renal histological specimens revealed no tubulointerstitial lesions in mice of either sex fed different diets. However, we observed Bowman’s space narrowing in glomeruli from WD-fed mice compared to age- and sex-matched SD-fed mice. Table 4 displays the qualitative, semi-quantitative grading of structural renal changes with respect to the proportion of glomeruli affected. Notably, according to the renal score grading used, a different pattern of frequency distribution between sexes was observed under WD: score 0: males, 14.28%, females, 0%; score 1: males, 42.85%, females, 85.71%; score 2: males, 48.85%, females, 14.28%.

Comparable histological features, with progressive collapse of Bowman’s space, have been described early in patients with diabetic nephropathy, leading to glomerulosclerosis in advanced stages of disease [79], and in rodent models fed a high-fat diet [80,81,82].

## 4. Discussion

Among available models of metabolic dysregulation, WD-fed C57Bl/6J mice are useful for studying the onset of diet-induced metabolic abnormalities by sex and age, contributing to the development of preventive measures and management strategies in biomedical research. In addition to overweight status, the proposed DIO animal model displays major metabolic alterations, such as dyslipidemia and insulin resistance, which are known to be associated with hepatic steatosis and an increased risk of cardiovascular and renal comorbidities. This multiorgan involvement has been characterized in a few studies on murine models of metabolic diseases [9,10], and HFUS appears an ideal tool to collect in vivo morphofunctional information on organs of interest in combination with conventional biochemical and histological techniques. In this study, we demonstrated that HFUS represents a useful complementary tool to monitor in vivo cardiovascular, hepatic, and renal changes in such animal models over time. Furthermore, our multimodality approach highlighted sex-specific differences in the C57Bl/6J DIO murine model fed with WD, mainly explained by the influence of sexual hormones, as in humans [83].

As expected, WD-fed mice of both sexes showed greater changes in BW gain than SD-fed mice as a result of higher caloric intake and FER. WD-fed males and females showed differences in fat distribution, as evidenced in vivo by higher BCS score, and at necropsy by a greater amount of subcutaneous and visceral adipose tissue, as well as a paler liver in males. Furthermore, both WD-fed males and females showed hepatomegaly, as evidenced by liver/body weight ratio results. In our study, non-fasting total cholesterol increased significantly in earlier experimental phases in both sexes as a response to WD. Furthermore, WD-fed mice demonstrated changes in glucose homeostasis and insulin sensitivity, as well as higher values of transaminases and lower BUN values compared to SD-fed mice of the same sex, which could likely reflect the involvement of diverse organs in diet-related metabolic changes, including heart, liver, and kidney, as detailed above [73,74,75,76,77,84]. In the present study, the effects of lipid overload in these organs were assessed in vivo and over time by HFUS, detecting early changes at 16 weeks of age for some parameters. Our results are indicative of early stages of cardiac remodeling, likely toward concentric hypertrophy in females and eccentric hypertrophy in males, with preserved ejection fraction associated with significantly different LVPW values in WD-fed male mice, as well as CO and RWT parameters in WD-fed female mice, from the age of 16 weeks [85,86]. Nevertheless, WD-fed female mice showed reduced SV and CO compared to their SD-fed counterparts, suggesting incipient progression to deterioration of systolic function [87]. Our findings appear consistent with those of previous studies, showing that large dietary amounts of fat and refined sugars contribute to early metabolic alterations in the myocardium, quite distinct from the chronic conditions of diabetic cardiomyopathy or cardiac hypertrophy induced mainly by fat overload [83]. Such alterations could be very interesting from a translational point of view, perhaps more closely resembling those that occur in humans and more easily reversible with therapies [85]. A previous study showed that in C57Bl/6J male mice feeding a WD for 24 weeks, diastolic dysfunction was detectable by echocardiography after 16 weeks of diet duration, followed by signs of systolic dysfunction starting from 20 and 24 weeks of diet [88]. Another recent report describes, in the same animal model fed a WD for 7 months, a distinct cardiac remodeling phenotype characterized by a reduction in EF and SV associated with alterations in cardiac metabolism due to an anomalous accumulation of lipids both in the endothelium and in the myocardium [89]. Considering this evidence, a strength of our study is that it included both sexes in the echocardiographic analysis, but a limitation was that diastolic function and strain analysis of the left ventricle were not performed. Similarly, HFUS allowed us to detect an increase in liver echogenicity and parenchymal heterogeneity at 16 and/or 24 weeks of age and that the brightness of liver parenchyma became comparable up to exceeding that of the right renal cortex at the intermediate or final experimental time point. The HR ratio showed a trend toward greater values in WD-fed male mice, reaching significance in WD-fed female mice compared to SD-fed counterparts. Moreover, the tissue heterogeneity was significantly higher in WD-fed mice compared to SD-fed mice of the same age and sex. Overall, we found concordance between qualitative ultrasound evaluation of liver changes and histopathological analysis of NAFLD at 24 weeks of age, as well as a significant correlation in WD-fed male mice between semi-quantitative HFUS visual grading, HR index, mean liver echogenicity, and NAFLD histological score. The absence of significant differences for some hepatic measurements obtained with HFUS between the matched groups could be related to technical limits of specificity and sensitivity in the case of fat accumulation in the liver was quite mild [11]. Furthermore, the heterogeneity of phenotypes induced in C57Bl/6J mouse substrain feeding high-fat dietary regimens, related to variability in individual response, might result in statistically non-significant results [90,91]. Furthermore, the quantitative and qualitative parameters used in our study were derived from various preclinical and clinical studies, but an ultrasonographic multiparametric analysis system for hepatic steatosis has not yet been standardized in mice [8,9,44,45,46,47]. Therefore, the absence of significance for some quantitative ultrasound characteristics could also be related to interspecies differences, suggesting the need for further investigations to implement more specific diagnostic approaches dedicated to murine species. Finally, HFUS provided morphological and functional information on the kidney in vivo, including information on the renal circulation, complementary to that obtained by traditional histology.

In our study, PI and RI were significantly reduced in WD-fed female mice compared to SD-fed ones. In the literature, PI and RI were found to be significantly correlated to glomerular filtration rate and serum creatinine and very sensitive to identify early stages of chronic kidney disease in case of obesity and diabetes [57]. Usually, increased RI suggests impaired renal function, although it could also indicate extra-renal pathologies [86,92]. The development of renal alterations after the consumption of a WD has been observed particularly in women, but the pathogenetic mechanisms involved are poorly understood [93]. The WD-induced hepato-cardio-renal syndrome has been investigated in rodent models, suggesting impaired activation of the renin-angiotensin aldosterone system, especially in female mice [93]. The complex interplay of different factors (WD, overweight, impaired glucose tolerance, dyslipidemia, fatty liver disease, and aging) could contribute to metabolic cardiomyopathy characterized by impaired diastolic relaxation with preserved ejection fraction, which in turn could induce a reduction in the LV early diastolic filling [86]. In this regard, due to the limitations of the available equipment/technical issues, the evaluation of diastolic function was not carried out by echocardiography, and serum creatinine was not determined in WD-fed mice, even if this serum parameter resulted within normal ranges in mice of both sexes fed SD, encouraging future research that addresses these gaps.

In conclusion, the reference values for the C57BL/6J mice of both sexes reported here could contribute to support researchers for interpreting the biological data of mouse mutants and corresponding wild-type controls based on the substrain examined. Overall, our findings showed that HFUS might be a useful tool to complement conventional methods of studying DIO research models for more comprehensive evaluation. HFUS offers several possibilities for refinement in preclinical research; for example, it does not use ionizing radiation and allows the examination of different organs with minimal discomfort for mice. In addition, HFUS provides longitudinal information on disease progression in the same subject, avoiding the sacrifice of cohorts of animals at different times, and improves statistical power compared to cross-sectional studies, allowing each animal to be its own control. Finally, ultrasound imaging is a relevant translatable imaging modality, which could improve the “bench-to-bedside” process, rapidly exploiting preclinical research findings into clinical research applications. Although the relatively low signal-to-noise ratio in ultrasound images represents a potential challenge in calculating image features compared to other imaging modalities, this is a rapidly evolving technology, and the development of advanced computerized image analysis methods will be crucial to improve diagnostic accuracy.

## Figures and Tables

**Table 1:** Descriptive statistics (mean ± SD) for male and female WT mice—Echocardiographic morphometric and functional analysis over the time in experimental groups: effects of WTD on cardiac remodeling and left ventricular (LV) contractility indexes overtime (8, 16 and 24 weeks of age). Abbreviations: M = male; F = female; d = diastole; s = systole; IVS/LVAW: interventricular septum/left ventricle anterior wall; LVPW: left ventricle posterior wall; LVID: left ventricle internal diameter; LV mass = left ventricle mass; LV vol = left ventricle volume; LV SV = left stroke volume; hr = heart rate, LV CO = left ventricle cardiac output; EF = left ventricle ejection fraction; FS = left ventricle fractional shortening; RWT = relative wall thickness. IVS/LVAW, LVID, LVPW are normalized for BW1/3; LV mass, LV vol, SV, CO are normalized for BW. Inferential statistical tests are described above in the text.

Age	Group	IVS/LVAW; d (mm)	IVS/LVAW; s (mm)	LVID; d (mm)	LVID; s (mm)	LVPW; d (mm)	LVPW; s (mm)	LV Mass Corr (mg)	
8 weeks	M SD	0.126 ± 0.020	0.175 ± 0.028	0.461 ± 0.037	0.333 ± 0.036	0.129 ± 0.027	0.175 ± 0.025	4.446 ± 0.755	
	M WD	0.095 ± 0.014	0.14 ± 0.023	0.48 ± 0.069	0.33 ± 0.054	0.104 ± 0.012	0.147 ± 0.021	3.42 ± 0.83	
16 weeks	M SD	0.112 ± 0.011	0.152 ± 0.017	0.347 ± 0.034	0.254 ± 0.018	0.123 ± 0.015	0.150 ± 0.023	4.19 ± 0.45	
	M WD	0.094 ± 0.015	0.14 ± 0.016	0.35 ± 0.060	0.23 ± 0.058	0.097 ± 0.015	0.130 ± 0.017	4.20 ± 1.13	
24 weeks	M SD	0.120 ± 0.013	0.159 ± 0.007	0.330 ± 0.035	0.23 ± 0.040	0.116 ± 0.017	0.146 ± 0.012	4.46 ± 0.69	
	M WD	0.092 ± 0.012	0.12 ± 0.019	0.32 ± 0.064	0.22 ± 0.076	0.088 ± 0.012	0.125 ± 0.034	3.75 ± 0.90	
8 weeks	F SD	0.141 ± 0.012	0.183 ± 0.028	0.541 ± 0.036	0.419 ± 0.057	0.137 ± 0.013	0.167 ± 0.025	4.44 ± 0.44	
	F WD	0.115 ± 0.021	0.173 ± 0.022	0.61 ± 0.084	0.43 ± 0.082	0.135 ± 0.017	0.190 ± 0.027	3.85 ± 0.92	
16 weeks	F SD	0.119 ± 0.015	0.176 ± 0.013	0.471 ± 0.032	0.31 ± 0.029	0.131 ± 0.014	0.182 ± 0.021	4.41 ± 0.52	
	F WD	0.126 ± 0.018	0.174 ± 0.029	0.447 ± 0.027	0.322 ± 0.051	0.146 ± 0.029	0.176 ± 0.028	4.31 ± 0.96	
24 weeks	F SD	0.139 ± 0.014	0.186 ± 0.017	0.38 ± 0.030	0.25 ± 0.019	0.144 ± 0.024	0.190 ± 0.008	4.52 ± 0.43	
	F WD	0.114 ± 0.023	0.168 ± 0.035	0.387 ± 0.024	0.267 ± 0.042	0.135 ± 0.040	0.180 ± 0.051	4.21 ± 1.03	
**Age**	**Group**	**LV vol; d (uL)**	**LV vol; s (uL)**	**LV SV (uL)**	**hr (bpm)**	**LV CO (mL/min)**	**EF (%)**	**FS (%)**	**RWT**
8 weeks	M SD	2.277 ± 0.381	1.04 ± 0.279	1.234 ± 0.195	497.71 ± 52.29	0.61 ± 0.13	54.67 ± 6.68	27.78 ± 4.17	0.557 ± 0.107
	M WD	2.60 ± 0.83	1.03 ± 0.39	1.56 ± 0.61	499.2 ± 25.45	0.77 ± 0.29	59.70 ± 10.01	31.49 ± 6.82	0.42 ± 0.08
16 weeks	M SD	1.63 ± 0.32	0.75 ± 0.10	0.87 ± 0.35	484.28 ± 42.14	0.43 ± 0.17	51.87 ± 13.26	26.34 ± 8.10	0.68 ± 0.12
	M WD	2.15 ± 0.85	0.83 ± 0.46	1.32 ± 0.49	420.7 ± 36.12	0.54 ± 0.16	63.04 ± 10.22	34.10 ± 7.21	0.54 ± 0.11
24 weeks	M SD	1.57 ± 0.39	0.73 ± 0.30	0.84 ± 0.19	501.42 ± 45.06	0.42 ± 0.10	54.41 ± 10.02	27.73 ± 6.36	0.73 ± 0.14
	M WD	1.80 ± 0.71	0.86 ± 0.62	0.94 ± 0.24	500.8 ± 46.25	0.47 ± 0.12	56.08 ± 17.84	30.23 ± 13.40	0.58 ± 0.12
8 weeks	F SD	2.56 ± 0.40	1.39 ± 0.44	1.17 ± 0.47	498.5 ± 38.58	0.58 ± 0.24	45.35 ± 15.57	22.52 ± 8.78	0.51 ± 0.055
	F WD	3.06 ± 0.81	1.32 ± 0.55	1.73 ± 0.35	389.6 ± 36.95	0.67 ± 0.14	57.77 ± 7.81	29.91 ± 5.39	0.41 ± 0.08
16 weeks	F SD	2.36 ± 0.34	0.93 ± 0.17	1.43 ± 0.34	471.87 ± 42.60	0.68 ± 0.18	60.20 ± 8.29	31.66 ± 5.75	0.53 ± 0.08
	F WD	1.99 ± 0.38	0.92 ± 0.38	1.07 ± 0.14	362.12 ± 75.27	0.39 ± 0.10	55.28 ± 11.57	28.32 ± 7.33	0.61 ± 0.12
24 weeks	F SD	1.61 ± 0.32	0.59 ± 0.11	1.01 ± 0.39	486 ± 31.88	0.49 ± 0.19	61.26 ± 12.42	32.50 ± 9.00	0.75 ± 0.15
	F WD	1.77 ± 0.26	0.75 ± 0.31	0.72 ± 0.30	407.2 ± 38.39	0.29 ± 0.12	59.11 ± 13.05	31.17 ± 9.02	0.64 ± 0.13

**Table 2:** US visual grading for male and female SD- vs. WD-fed mice: effects of diet intervention on liver echostructure and echogenicity in sex- and age-matched SD- vs. WD-fed mice at 8, 16 and 24 weeks of age (each box reports the number of mice showing a specific HFUS score for each time point). Abbreviations: M = male; F = female.

US Findings/Time of Experiment	SD M Mice			WD M Mice		
	8 Weeks (n = 7)	16 Weeks (n = 7)	24 Weeks (n = 7)	8 Weeks (n = 8)	16 Weeks (n = 8)	24 Weeks (n = 8)
Homogeneous liver parenchyma of medium level echogenicity	7	7	6	8	0	0
Diffusely increased parenchymal echogenicity	0	0	1	0	8	1
Discrete coarsened and heterogeneous parenchyma	0	0	0	0	0	6
Extensive coarsened and heterogeneous parenchyma	0	0	0	0	0	1
L-Echo < R-Echo	7	7	7	8	7	1
L-Echo = R-Echo	0	0	0	0	1	5
L-Echo > R-Echo	0	0	0	0	0	2
Presence of Ascites	0	0	0	0	0	0
US findings/Time of experiment	SD F mice			WD F mice		
	8 weeks (n = 8)	16 weeks (n = 8)	24 weeks (n = 8)	8 weeks (n = 8)	16 weeks (n = 8)	24 weeks (n = 8)
Homogeneous liver parenchyma of medium level echogenicity (pattern 1)	8	8	8	8	0	0
Diffusely increased parenchymal echogenicity (pattern 2)	0	0	0	0	8	5
Discrete coarsened and heterogeneous parenchyma (pattern 3)	0	0	0	0	0	3
Extensive coarsened and heterogeneous parenchyma (pattern 4)	0	0	0	0	0	0
L-Echo < R-Echo	8	8	8	8	7	2
L-Echo = R-Echo	0	0	0	0	1	6
L-Echo > R-Echo	0	0	0	0	0	0
Presence of Ascites	0	0	0	0	0	0

**Table 3:** Histological characteristics of murine liver samples per score grading category (each box reports the number of mice showing a specific NAFLD score). Abbreviations: M = male; F = female.

Group	Histological Features Scoring System																
	SAF Score Grading: Percentage of the Total Area Affected			NAFLD Score Grading: Percentage of the Total Area Affected [Macrovescicular (Score 0–3), Microvescicular (0–3), Hypertrophy (0–3); Inflammation (0–3)].										Fibrosis Score Grading: Qualitative/Semiquantitative Visual Evaluation			
	0	1	2	0	1	2	3	4	5	6	7	8	9	Absent	Mild	Moderate	Severe
SD M (n = 7)	2	4	1	7	0	0	0	0	0	0	0	0	0	7	0	0	0
WD M (n = 8)	0	0	8	3	1	1	3	0	0	0	0	0	0	4	4	0	0
SD F (n = 8)	0	8	0	8	0	0	0	0	0	0	0	0	0	8	0	0	0
WD F (n = 8)	0	0	8	1	2	2	3	0	0	0	0	0	0	6	2	0	0

**Table 4:** Histological characteristics of murine kidney samples per score grading category (each box reports the number of mice showing a specific histological score). Abbreviations: M = male; F = female. * missing data.

Group	Histological Features Scoring System														
	Renal Score Grading: (Percentage of the Glomeruli Altered, %)			Bowman’s Capsule and Space Score Grading: Percentage of the Glomeruli with Narrowed/Collapsed Bowman’s Space (%)											
	0 (<30)	1 (30–70)	2 (>70)	0	1–1–5	6–10	10–15	15–20	21–25	16–30	31–40	41–50	51–60	61–70	>70
SD M (n = 7)	7	0	0	1	3	2	1	0	0	0	0	0	0	0	0
WD M (n = 7) *	1	3	3	0	0	0	0	0	1	0	2	1	0	0	3
SD F (n = 8)	8	0	0	1	2	1	4	0	0	0	0	0	3	0	0
WD F (n = 7) *	0	6	1	0	0	0	0	0	0	0	1	1	3	1	1

## Data Availability

The raw data and digital data supporting the conclusions of this article are available upon request from the corresponding author because the data are part of an ongoing study and/or due to technical and time limitations.
